# Unraveling tumor microenvironment heterogeneity in malignant pleural mesothelioma identifies biologically distinct immune subtypes enabling prognosis determination

**DOI:** 10.3389/fonc.2022.995651

**Published:** 2022-09-27

**Authors:** Kaidi Yang, Tongxin Yang, Tao Yang, Ye Yuan, Fang Li

**Affiliations:** ^1^ Department of Oncology, Hainan Hospital of Chinese People’s Liberation Army General Hospital, Sanya, China; ^2^ Institute of Pathology and Southwest Cancer Center, Southwest Hospital, Third Military Medical University (Army Medical University), Chongqing, China

**Keywords:** malignant pleural mesothelioma, immune subtypes, immunotherapy, prognosis, machine learning-based gene classifier

## Abstract

**Background:**

Malignant pleural mesothelioma (MPM) is a rare and intractable disease exhibiting a remarkable intratumoral heterogeneity and dismal prognosis. Although immunotherapy has reshaped the therapeutic strategies for MPM, patients react with discrepant responsiveness.

**Methods:**

Herein, we recruited 333 MPM patients from 5 various cohorts and developed an *in-silico* classification system using unsupervised Non-negative Matrix Factorization and Nearest Template Prediction algorithms. The genomic alterations, immune signatures, and patient outcomes were systemically analyzed across the external TCGA-MESO samples. Machine learning-based integrated methodology was applied to identify a gene classifier for clinical application.

**Results:**

The gene expression profiling-based classification algorithm identified immune-related subtypes for MPMs. In comparison with the non-immune subtype, we validated the existence of abundant immunocytes in the immune subtype. Immune-suppressed MPMs were enriched with stroma fraction, myeloid components, and immunosuppressive tumor-associated macrophages (TAMs) as well exhibited increased TGF-β signature that informs worse clinical outcomes and reduced efficacy of anti-PD-1 treatment. The immune-activated MPMs harbored the highest lymphocyte infiltration, growing TCR and BCR diversity, and presented the pan-cancer immune phenotype of IFN-γ dominant, which confers these tumors with better drug response when undergoing immune checkpoint inhibitor (ICI) treatment. Genetically, BAP1 mutation was most commonly found in patients of immune-activated MPMs and was associated with a favorable outcome in a subtype-specific pattern. Finally, a robust 12-gene classifier was generated to classify MPMs with high accuracy, holding promise value in predicting patient survival.

**Conclusions:**

We demonstrate that the novel classification system can be exploited to guide the identification of diverse immune subtypes, providing critical biological insights into the mechanisms driving tumor heterogeneity and responsible for cancer-related patient prognoses.

## Introduction

Malignant pleural mesothelioma (MPM) is a rare and lethal cancer arising from the linings of the lungs, known as the pleura (1). Due to its insidious onset and high local invasiveness, this cancer is often diagnosed at an advanced stage, rendering it incurable (2).

For a long time, platinum-based chemotherapy combined with pemetrexed has been the state-of-the-art treatment for advanced MPM (3). Drug development for this lethal cancer has been slowly pushed over the last two decades until the recent advances in immune checkpoint inhibition (4, 5). Immune checkpoint inhibitors (ICIs) targeting PD-1 and CTLA4 have shown encouraging clinical activity with good tolerability in untreated, histologically confirmed unresectable MPMs relative to standard first-line chemotherapy. Nonetheless, the median life expectancy is only one and a half years, even with 4-months extended survival benefits (6). The varying clinical responses to ICIs and no credible biomarkers available emphasize a more personalized regimen for MPMs (7).

Overexpression of PD-L1 has been confirmed as a predictor of response to anti-PD1 therapy in multiple solid tumors, whereas efficacy by PD-L1 status demonstrated no improvements in survival benefits for MPM patients (7, 8). Emerging evidence indicated the intra-tumoral CD8+ T cell infiltration, TGF-β signaling, and Treg content were associated with curative responses and outcomes (9–11), while for MPM, limited research was available. MPM develops in a heterogeneous immune microenvironment that dynamically interacts with mesothelioma tumor cells to sustain cancer growth and progression (12–14). We hypothesized that a deep dissection of the immunological profiles within MPMs would provide a framework for an in-depth understanding of the immune-genomic mechanisms responsible for cancer-related prognosis and maximize response to immune-based therapies. Lee et al. recently profiled the intratumoral cellular networks within 12 MPMs using CyTOF and defined two immunologic subtypes showing predictive value for ICI response (15). However so far, there is no extensive cohort-based immunological classification system for MPMs, and a robust gene classifier specific for predicting prognosis and subtyping is still lacking.

In this work, we enrolled 333 MPM patients from five independent cohorts as a large-sample MPM cohort, and 87 MPM patients came from TCGA dataset. Several unsupervised classification methods, particularly the non-negative matrix factorization (NMF) and nearest template prediction (NTP) algorithms, were applied to distinguish distinct immunological phenotypes and reveal the intratumoral heterogeneity of MPMs. The predictive, reliable multi-gene classifier holds the value in immune subtyping and prognostic determination and can be used to guide immunotherapy strategies.

## Methods

### Malignant pleural mesothelioma patient cohort

We enrolled the gene expression profiles and clinical information of MPM datasets from Gene Expression Omnibus (GEO) with the accession numbers GSE29354 (16), GSE2549 (17), GSE163722 (18), and GSE51024 (19). The expression files of the MTAB-6877 dataset (20) were provided in ArrayExpress (https://www.ebi.ac.uk/arrayexpress/). The *ComBat* method came from *sva* package (R version 3.38.0) was used to remove batch effects across different microarray platforms. As shown in **Figure S1A**, the deviations of mean gene expression were removed and the five datasets were thus comparable to each other. Subsequently, a large MPM dataset including 333 qualified expression profiles was set as a training cohort, while the RNA-seq v2 level-3 dataset of TCGA-MESO (Mesothelioma, from UCSC-Xena) was used for external validation. Detailed information on these datasets is shown in **Supplementary Table S1**. To validate the finding in proteomic level, we performed extensive analysis of the Reverse Phase Protein Array (RPPA) data from TCGA cohort on 63 human MPMs characterized with a set of 219 protein features.

### NMF classification

After reserving high-variance gene features ranked in the top half of total samples, we performed subtype classification with the mRNA expression profiles using the NMF algorithm packed into the *NMF* package (R version 0.24.0). We plotted the rank-changing trend diagram of the cophenetic coefficient and determined the point that dropped the most along with the rank-changing as the best rank (number of classification). To functionally annotate each subclass/module, the gene signatures were extracted using the *extractfeatures* function and subsequently used for gene over-representation (ORA) analysis utilizing the *clusterProfiler* package (R version 4.5.0). MPMs conferred with the highest immune module score were denoted as an immune-related subtype. Then, the top 200 exemplar genes in the immune module were identified as the classifier genes to dichotomize samples into the immune and non-immune subtypes, further optimized by the multidimensional scaling random forest (MDS-RF) algorithm. To sub-classify immune MPMs, a 26-immune signature scoring file was generated from the *IOBR* package (R version 0.99.0) as an input into the NTP module (GenePattern platform, https://cloud.genepattern.org). The molecular similarity between the two MPM cohorts was estimated using Subclass Mapping (GenePattern).

### Immune signature analysis

To delineate the tumor microenvironment (TME) contexture, the *IOBR* package (https://github.com/IOBR/IOBR) integrating eight published methodologies was used for computing the single-sample gene set enrichment (ssGSEA) score (21). Identifying TME signatures associated with ICI response was performed using the *iobr_cor_plot* function. Immune-related indices, previously defined by TCGA pan-cancer programs, were incorporated into comparisons across different subtypes. Also, Thorsson’s pan-cancer immune phenotyping (22) was used for feature comparisons and subtype correlations. Immune-related indices, including Stroma fraction, Leukocyte fraction, TCR richness, and so on, were obtained from supplementary material of Thorsson’s research. Of note, the index has been adjusted for tumor purity as demonstrated. Tumor immune proportions were computed by CIBERSORT, which ran with mRNA profiles as input and produced absolute abundances of 22 immune components. To predict the immunotherapy response of MPM patients, we imported the tumor pre-treatment expression profiles into TIDE (http://tide.dfci.harvard.edu), which computed the response scores based on signatures of T-cell dysfunction and exclusion, the two primary mechanisms of tumor immune evasion (23). The cohort of human MPMs that received anti-PD-1 immunotherapy (GSE99070) was considered for investigating of association between immune subtypes and immunotherapy response.

### Pathway activity analysis

The dataset of pathway activity comprising 1387 constituent pathways was downloaded from UCSC-Xena browser (http://xena.ucsc.edu/). Pathway analysis was conducted using the PARADIGM algorithm (24), and the top expressed pathways were generated through differential expression analysis.

### Genomic mutation analysis

The mutation annotation format (MAF) file with aggregated somatic mutation annotation of TCGA MPM cases was deposited in the TCGA portal. For summarization, analyses, and visualization of somatic genomic alterations, various functions were provided by the *maftools* package (R version 2.7.40). The *mafCompare* function was applied to compare two groups to identify and visualize differentially mutated genes. The *clinicalenrichment* function was used for groupwise comparisons, thus identifying enriched mutations or copy number variations (CNVs) for each subtype. We ran MutSigCV1.41 using the recommended default parameters on GenePattern to identify the driven mutations highly relevant to MPMs (q-value < 0.10).

### Subtype classifier identification

First, we prefiltered genes by different feature selection algorithms, including *Chi2-algorithm*, *Fast correlation-based filter*, and *Information gain* using the *Biocomb* package (R version 0.4). This set of gene candidates was supplemented with gene features computed by machine-learning models of *Randomforest* (RF), *XGBoost*, and *Brutal*. Genes nominated by multiple algorithms were ranked by the frequency of being selected by the six methods. It resulted in a panel of 94 genes selected by at least four algorithms. (**Supplementary Table 2**) Then, we used the *findCorrelation* function packed in the *Caret* package 6.0-92 to remove highly correlated features. If two features have a high correlation, the function looks at each feature through pair-wise correlations and removes the feature with the mean absolute correlation over 0.8. After removing features showing high correlation, we adopted a stepwise selection strategy to determine the optimal size of the gene panel. Specifically, starting from the top-ranked gene (ordered by count and index of mean decrease accuracy), gene panels with incremental sizes (adding one gene at a time) were evaluated for their ability to correctly classify each case by RF with a cross-validation approach of LOOCV (leave-one-out cross-validation). To identify the best feature combination to improve classification accuracy, especially for distinguishing immune-activated from immune-suppressed subtype, we adopted the approach similar to the multiple algorithms-based feature selection method above, followed by removing features with high pair-wise correlations. The two comparisons (immune versus non-immune, immune-activated versus immune-suppressed) respectively generate seven and nine genes through the *recursive feature elimination* (RFE) algorithms using *rfeControl* functions (from *Caret* package 6.0-92), which was assisted by machine learning methods of RF-LOOCV or RF-CV. The optimal features computed from two classification systems were combined into a 16-gene panel for the following filtering step. All the models metrics of gene features filtering and selection process were stored in **Supplementary Table 3**. Then, we apportion the data into training and test sets, with 70-30 splits, and fit the models on the training sets. By evaluating the performance of different machine learning models (Linear discriminant analysis, Naive Bayes, Bagged trees, and RF with LOOCV or CV) on testing sets, we identified the best machine-learning model (with the highest prediction accuracy) when undergoing the RFE process, which generated the optimal gene features. The selected gene classifier was evaluated for its predictive efficiency in the external TCGA-MESO dataset.

### Statistical analysis

When the dependent variable was continuous but not normally distributed for two independent groups, the Mann-Whitney U test was used for comparisons. In comparison, normally distributed data were compared between two groups *via* the Student t-test. We performed a Shapiro test to check whether the considered data is normally distributed data or not by the *stats* package (R version 4.0.4). Kaplan-Meier plot and Log-rank test were used to estimate the survival curve and compare the difference in survival curves between different groups. The Chi-square test illustrated the correlations between newly defined subtypes and proposed molecular subtypes. The forest plot was used to visualize the prognostic impact of individual variables of the multivariate Cox regression model using the *forestmodel* package (R version 0.6.2). All analyses were performed by Graphpad Prism 8.0 or R version 4.0.2, and a two-sided *p-*value less than 0.05 was considered statistically significant.

## Results

### Identification of immune-associated subtype for an integrated large-sample MPM cohort

**Figure 1:**
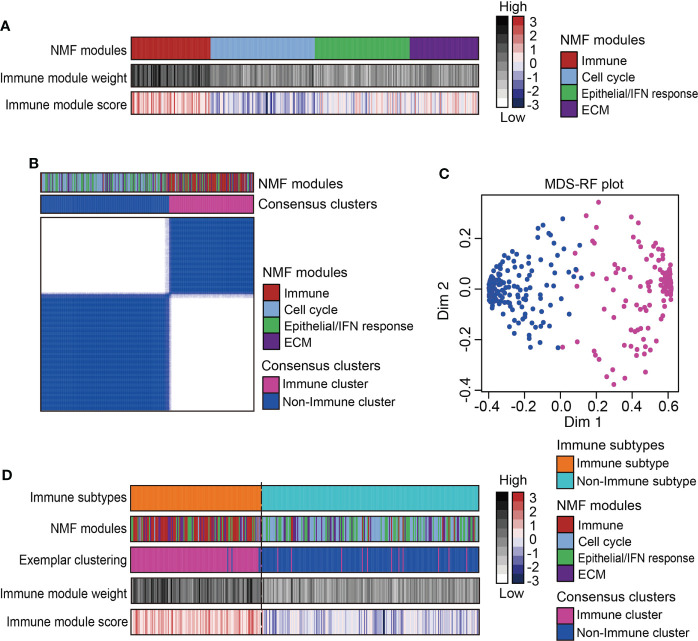
Identification of immune subtype for an integrated large-sample MPM cohort. **(A)** Non-negative matrix factorization (NMF) algorithms identified four functional expression modules to classify the microarray-based expression profiles of 333 MPM samples. One expression module showed the highest immune enrichment score and NMF module weight was marked red and recognized as an immune-associated subtype. IFN, Interferon; ECM, Extracellular matrix. **(B)** Consensus clustering based on the top 200 exemplar genes expression identified two subclasses with one subclass presenting enrichment of immune-related NMF module. **(C)** The multidimensional scaling random forest (MDS-RF) refined the classification and divided whole MPM samples into immune and non-immune subtypes. **(D)** Heatmap shows the final classification results along with various NMF modules, exemplar clustering subtypes, immune module weight, and immune enrichment score.

A total of 333 MPMs patients from five independent cohorts were enrolled, along with their clinical information and microarray-based expression profiles. After correcting batch effects, an integrated large-sample MPM cohort were established for subsequent analysis. To obtain a robust classification system and distinct molecular patterns, we applied the NMF algorithm to reduce data dimensionality by decomposing it into several smaller non-negative factors with physical interpretation for subclass discovery. As cophenetic correlation coefficients from k = 2 to k = 10, we determined k=4 as the parameter that yielded the most robust clustering (**Figures S1B, C**). Among the four subclasses, we defined the one characterized with high immune enrichment scores as an immune-associated subtype, whereas the other three subclasses were respectively termed as Cell cycle-, Epithelial/Interferon (IFN) response-, and Extracellular matrix (ECM)-related subtypes according to the results of ORA analysis (**Figure 1A** and **Figure S2**). The top 200 weighted genes in the immune module/subclass were defined as exemplar genes that reflect the core features of immune components in MPM (**Figure S1D**). To simplify the subtyping process for fast-recognition of immune related subtypes, we performed consensus clustering analysis using the exemplar genes, which classified MPM patients into immune and non-immune subclasses (**Figure 1B**). Next, this classification was further modified by the MDS-RF algorithm (**Figure 1C**). The sorting result of multiple methods for the 333 MPM patients was shown in **Figure 1D** and **Supplementary Table 4**. We presented that simplifying the classification process using the top 200 weighted genes matched with the genome-wide expression profiling-based NMF algorithm for identifying immune-related subtypes for MPMs.

### Sub-classification and dissection of MPM immune microenvironment

**Figure 2:**
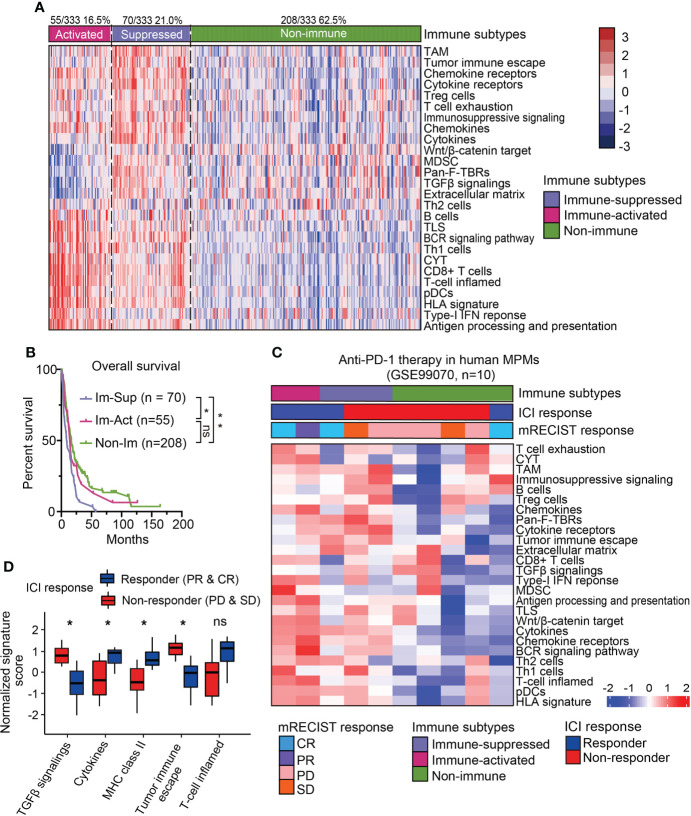
Sub-classification and dissection of MPM immune microenvironment. **(A)** The immune subtype was further subdivided into immune-activated (70/125, 21.0%) and immune-suppressed (55/333, 16.5%) MPMs, using nearest template prediction (NTP) analysis with signatures covering 26 immune- and TME-related signatures. TAM, Tumor-associated macrophage; MDSC, Myeloid-derived suppressor cell; TLS, Tertiary lymphoid structure; CYT, Cytolytic activity. pDCs, Plasmacytoid dendritic cell; Pan-F-TBRs, Pan fibroblast TGF-β response signature. **(B)** Kaplan-Meier survival analyses of overall survival in the integrated MPM cohort with different immunological subtypes. **, *p* < 0.01; *, *p* < 0.05; n.s., Not significant. **(C)** The heatmap showing the expressions of 26 immune- and TME-related signatures in the pretreatment human MPM cohort upon the anti-PD-1 treatment. The dataset was classified using the same approach as previously shown in **Figure 2A**. Top column shows the corresponding immune subtype and mRECIST response of each case. ICI, Immune checkpoint inhibitor. **(D)** The normalized signature score of immune characteristics in anti-PD-1 response and non-response subgroups. CR, Complete response; PR, Partial response; PD, Progression disease; SD, Stable disease.

Recent studies on the immunological microenvironment revealed three representative phenotypes with general applicability, including inflamed, excluded, and desert subtypes (25). To further dissect the immunological heterogeneity for MPMs, scorings of 26 immune-related signatures were collected to subdivide the immune-related MPMs into two subsets using the NTP algorithm. One subset of 55 patients (16.5%, 55/333) showed increased enrichment of immunocytes, cytolytic activity (CYT) score, ooand IFN related signatures as compared with other MPMs and hence, was termed an immune-activated subtype (**Figure 2A**). Other indices like Wnt/β-catenin signaling, TGF-β signaling, and the extracellular matrix (ECM) have been shown to play an essential role in establishing immunological tolerance (9, 26, 27). Likewise, the myeloid components, including tumor-associated macrophages (TAMs) and myeloid-derived suppressor cells (MDSCs), act as central regulators of immune suppression and can secrete multiple soluble cytokines and chemokines to deactivate the process of immune activation (28). For these reasons, we defined the sub-classified subtype with high stroma infiltration and immune-suppressive components as immune-suppressed MPMs (21.0%, 70/333) (**Figure 2A**). The redefined three subtypes (immune-activated, immune-suppressed and non-immune) were relatively well distributed in different enrolled MPM cohorts (**Supplementary Table 5**). Histologically, consistent with previous finding (20), sarcomatoid MPMs was enriched in immune-activated MPMs relative to other subtypes (27.3% *versus* 3.9%, 12.1%). By contrast, greater proportions of epithelioid and biphasic tumors were respectively present in non-immune and immune-suppressed MPMs (**Figure S3A**). Next, we performed survival analysis and observed that immune-suppressed MPM patients displayed shortened survival relative to immune-activated or non-immune MPM patients (**Figure 2B**), indicating the prognostic significance of the immunological subtyping. The immune-related signature was a good indicator of patient survival (29). Our multivariate analysis by the Cox proportional hazards model on prognoses of patients indicated that the Th2 cells, MDSC, and Pan-F-TBRs (Pan fibroblast TGF-β response signature) informed poor outcome (**Figure S3B**). To investigate whether the immunological subtyping can predict the treatment response of ICIs, the pretreatment human MPMs (n=10) upon the immunotherapy of anti-PD-1 were classified using the same approach (GSE99070, **Figure 2C**). Intriguingly, although with only two patients, immune-activated MPM patients show partial or complete response to the treatment. By comparison, most of the immune-suppressed and non-immune patients (2/3, 4/5) were shown to be unresponsive when undergoing such therapy (**Figure 2C**), highlighting the relevance of our immunological subtyping to the therapeutic effects of ICI. Relative to the non-response subgroup, the ICI response subgroup showed prominent expressions of pathways regulating T-cell inflamed, cytokines, and MHC class-II, along with decreased enrichments of TGF-β signaling and tumor immune escape (**Figure 2D**). Immune-activated MPMs were enriched with these signatures and also highly expressed multiple immune checkpoint genes (**Figure 2A** and **Figure S4A**), demonstrating that ICI therapy is poised for clinical evaluation for them.

### Validation of the novel immune-subtyping in external TCGA-MPM cohort

**Figure 3:**
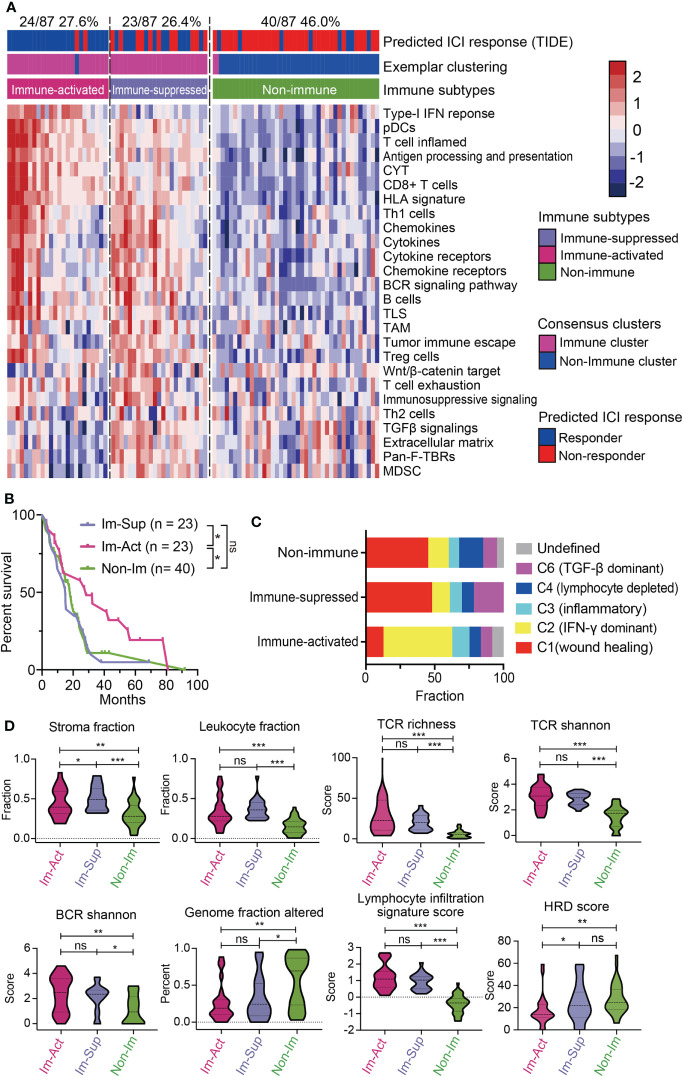
Validation of the novel immune-subtyping in external MPM cohort. **(A)** The TCGA-MPM cases were subdivided into non-immune (40/87, 46.0%), immune-suppressed (23/87, 26.4%), and immune-activated (24/87, 27.6%) MPMs using methods of consensus clustering, MDS-RF modification, and NTP division with signatures covering 26 immune- and TME-related signatures which were shown in the heatmap. Top column shows the predicted ICI response estimated by IOBR and corresponding immune subtype of each case. **(B)** Kaplan-Meier survival analyses of overall survival in MPMs with different immunological subtypes using TCGA RNA-seq cohort. **, *p* < 0.01; *, *p* < 0.05; n.s., Not significant. **(C)** Percentage column plots showing the distribution of predicted Thorsson’s pan-cancer immune phenotyping across the three immune subtypes. ***, *p* > 0.001; **, *p* > 0.01; *, *p* > 0.05; n.s., Not significant. **(D)** Scoring or fraction of immune cell components and indices in different immune subtypes.

To see whether the above classification system can be reappeared, we explored the expression profiles from the TCGA-MESO dataset containing 87 patients and performed the same classification procedures (**Figures S5A, B**). The classification results showed that 54.0% (47/87) of patients were immune-related MPMs characterized with distinct immune phenotypes. Among them, 24 patients (27.6%) were defined as immune-activated MPMs, and other 23 patients (26.4%) were regarded as immune-suppressed MPMs, enriched with TAMs, Treg cells, MDSCs and, tumor immune escape signatures, indicating that immune characteristics can reappear in the validation cohort (**Figure 3A**). Subtypes of the TCGA dataset showed high consistency with corresponding subtypes of the large-sample MPM cohort through subclass correlation analysis (**Figure S5C**), suggesting a good reproducibility of the three-subgroup-clustering system for identifying MPM immunological signatures. Kaplan-Meier survival analyses confirmed that immune-activated MPMs exhibited the most favourable outcome relative to the other two subtypes (**Figure 3B**). By associating our immune subtyping with Thorsson’s pan-cancer immune phenotyping (22), we found that approximately 50% of immune-activated MPMs pertained to the IFN-γ-dominant (C2) phenotype. In contrast, the wound healing (C1) phenotype occupied the most parts (47.8%, 45%) for the other two subtypes. C6 phenotype, defined as TGF-β dominant, showed the largest proportion in immune-suppressed MPMs compared with other subtypes (21.7% *versus* 10%, 8.3%) (**Figure 3C**), supporting the previous findings of TGF-β’s role in immunosuppression (30). Further profiling of the immune milieu demonstrated that three MPM subtypes manifested distinct immune-related signatures. Immune-activated MPMs harbor the lowest genomic alteration fraction and homologous recombination defects (HRDs), reflecting their ability to repair DNA damage and maintain genomic stability. The much more hypervariable and diverse TCR and BCR, and highest lymphocyte infiltration score informed increased probability of immune responsiveness to ICIs (**Figure 3D**), consistent with the findings of ICI response estimated by TIDE (**Figure 3A**). By contrast, immune-suppressed subtype MPMs displayed the highest stroma and leukocyte fractions (**Figure 3D**). Microsatellite instability (MSI) status and tumor mutation burden (TMB) has been widely recognized as biomarkers predicting the therapeutic efficacy of ICIs (31–33). As the ICI therapeutic effects estimated by TIDE (**Figure 3A**), the decreased MSI scores in immune-suppressed MPMs also informs poor ICI efficacy (**Figure S5D**). In comparison, as we observed, there was no significant difference in TMB levels across the three groups (**Figure S5D**).

Then, we investigated the oncogenic pathways mediating the distinct phenotype of each subtype. Interestingly, immune-activated MPMs displayed the most intense T cell-mediated antitumor response, with high scorings of the T-cell receptor, JAK-STAT, and interferon-γ. Stromal-enriched immune-suppressed MPMs were associated with high activities of MAPK, TNFα-NF-κB, and IL-7 signalings, while non-immune MPMs were characterized by abundant intracellular signals of N-cadherin, FGF, EphA2, EGFR, and hypoxia (**Figure S6A**).

### Genomic landscape of the three MPM immune subtypes

**Figure 4:**
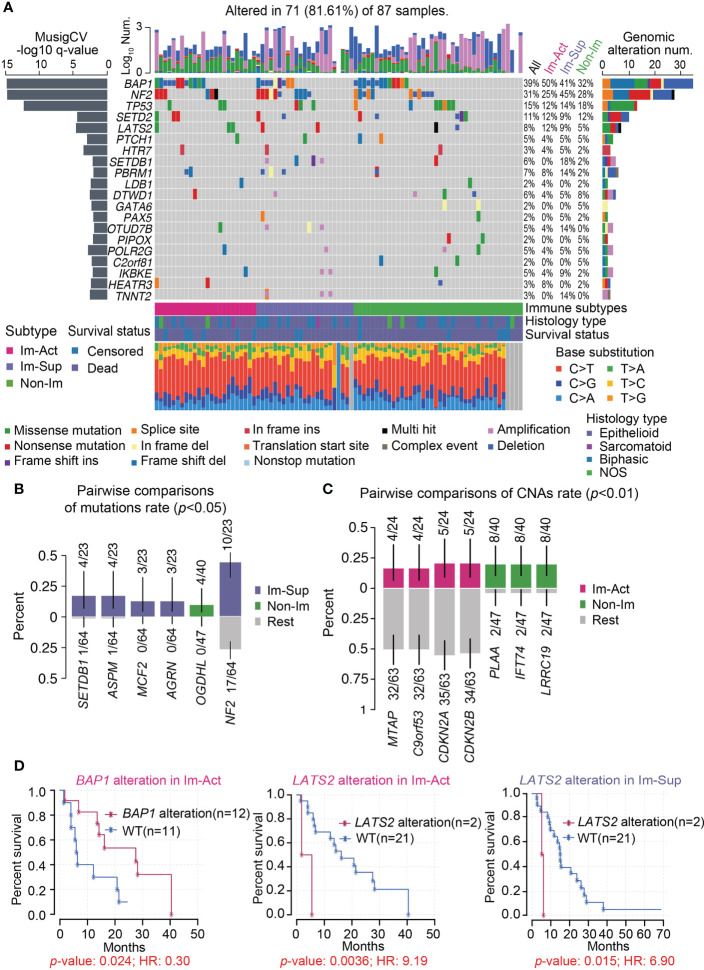
Genomic landscape of the three MPM immune subtypes. **(A)** Oncoplot representation of the distribution of genomic alterations (mutations and copy number variations) in driven genes identified by Mutsig across the three MPM immune subtypes, with the significance of mutations (Log10 transformation of MutSig q-value) shown at the left panel. Genomic alterations frequency of all MPM samples stratified by immunological subtypes were listed on the right side of the Oncoplot. The top column illustrates the overall counts of genomic alterations per sample with Log10 transformation, and the column at the bottom presents the mutation spectrum of base substitutions. NOS, Not otherwise specified. Del, Deletion. **(B, C)** Identification of significantly enriched mutations **(B)**, *p* < 0.05) or CNAs (copy number alterations) **(C)**, *p* < 0.01) of genes for each subtype by pairwise comparisons. The upper and bottom columns indicate the alteration rate in enriched subtype and the other subtypes respectively. **(D)** Kaplan-Meier curves of the overall survival in the corresponding subtype of MPM patients stratified by *BAP1* or *LATS2* genomic alteration status. WT, Wild-type.

Gene- or pathway-level somatic mutations were shown to affect the immune microenvironment. With the implementation of well-established statistical and computational methods in the *maftools* package, we presented the genomic landscape of mutational alterations and copy number variations across the three immunological subtypes. It is illustrated that the three immunological subtypes displayed distinct genomic characteristics (**Figure 4A** and **Figures S7A, B**). Of note, immune-suppressed MPMs had the highest genomic alteration rate of *SETDB1* and *NF2* (18%, 45%) relative to immune-activated (0%, 25%) and non-immune (2%, 28%) MPMs (**Figures 4A, B**). *BAP1* alteration was most commonly found in patients with immune-activated MPMs (**Figure 4A**) and was specifically associated with a favorable outcome for these patients (**Figure 4D** and **Figure S7C**), indicating subtype-specific prognostic value. *MTAP* loss is a reliable surrogate for *CDKN2A* (p16) homozygous deletion in mesothelioma diagnosis (34, 35). These two highly specific markers for malignancy lesions of mesothelioma have lower copy number deletion rates in immune-activated MPMs than other MPMs (**Figure 4C** and **Figure S7A**). *LATS2* mutation or inactivation is a positive regulator of mesothelioma proliferation *via* constitutively activating YAP and Hippo signaling pathways (36). Herein, we demonstrated that *LATS2* genomic alteration was an indicator of adverse prognosis for both immune-activated and immune-suppressed MPMs (**Figure 4D**), while the same finding was not observed in non-immune MPMs (**Figure S7D**). To support the findings at the proteomic level, we determined the phosphorylation levels of the residues serine 127 (S127) of YAP, together with a common *CDKN2A* encoding tumor suppressor, p16 (INK4A) (37), across the three subtypes using TCGA-RPPA dataset. As expected, immune-activated MPMs exhibited compromised phospho-YAP (S127) levels and upregulated p16 (INK4A) levels relative to non-immune MPMs with statistical significance, which would be a partial interpretation of the optimistic outcomes (**Figure S7E**).

### Development and validation of robust classifiers for distinguishing three subtypes of MPMs

**Figure 5:**
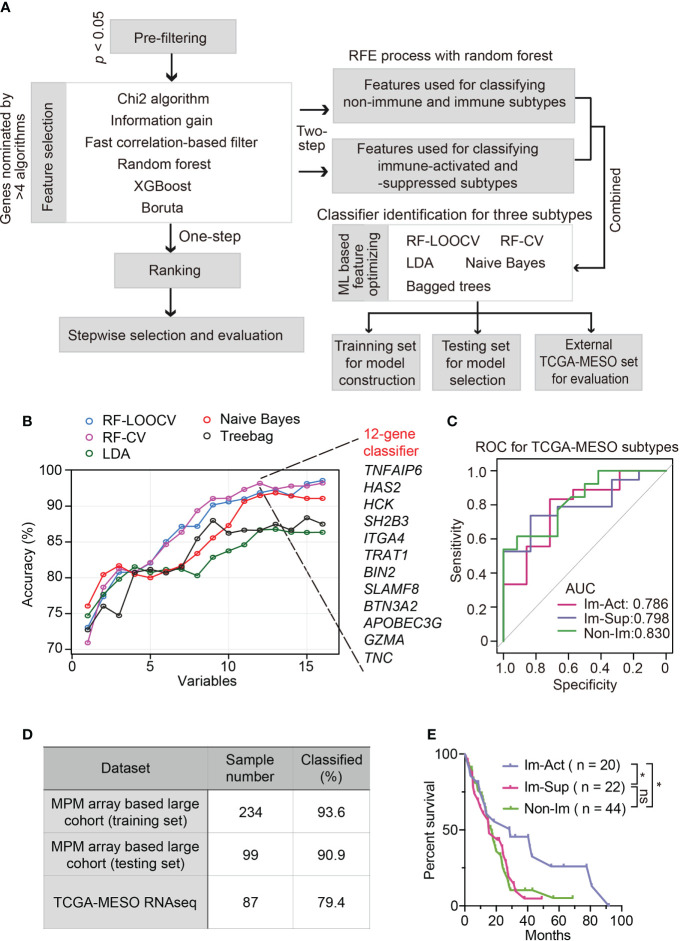
Development and validation of robust classifiers for distinguishing three subtypes of MPMs. **(A)** The workflow of a multi-step procedure for identifying classifiers to distinguish three immune subtypes of MPMs. LDA, Linear discriminant analysis; CV, Cross-validation; LOOCV, Leave-one-out cross-validation; ML, Machine learning. **(B)** Line graphs illustrate the variation trend of classification accuracy computed by multiple feature selection algorithms plus stepwise recursive feature elimination (RFE) process. The x-axis suggested a different number of variable combinations. **(C)** Receiver operating characteristic (ROC) curves of the 12-gene classifier for classifying each immune subtype separately. The scores of the area under curve (AUC) are presented in the plot. **(D)** Percentage of correctly classified samples using the 12-gene classifier in different MPM datasets. **(E)** Kaplan-Meier analysis of overall survival of TCGA MPM patients based on classification predicted by the 12-gene classifier. *, p < 0.05; n.s., Not significant.

To simplify a biomarker set classifying MPMs for molecular diagnosis and clinical practice, we set out to develop a robust panel of classifier genes with the application of machine-learning algorithms. The workflow is shown in **Figure 5A**. To identify non-redundant and uncorrelated marker genes, we assembled multiple variable feature-selection algorithms to select the most informative features and ranked the candidates in order of feature importance (**Supplementary Table 2**, see Materials and Methods for details). To determine the optimal gene panel size, we iteratively trained the RF-LOOCV model by adding one gene in one run. We noticed that classifier performance was almost no more improved for panels larger than 20 genes (**Figure S8A**). Although the average overall accuracy of classification reaches a maximum around 0.850, the capability of this model for distinguishing immune-activated MPMs from immune-suppressed MPMs is still far from satisfactory (**Figure S8A**). Therefore, we adopted a two-step feature selection process accompanied by an RFE algorithm to obtain the optimum gene combinations for improving the separating capacity of two comparisons, including immune *v.s.* non-immune and immune-suppressed *v.s.* immune-activated (**Figure S8B**). Using the combined 16-gene panel as an input for multiple machine learning training procedures, we identified the RF plus cross-validation (CV) algorithm as the best one in terms of classification accuracy. In this setting, a 12-gene classifier showed the highest discriminant performance (93.6%) with RFE process on the training set and was thus identified as the best optimal set (**Figure 5B**). The efficiency of this 12-gene classifier was confirmed using a testing set and external TCGA dataset with accuracies of 90.9% and 79.4% (**Figure 5D**). More importantly, using the 12-gene classifier, each immune subtype can be efficiently diagnosed with no bias (**Figure 5C**). The summarization of the feature selection process was shown in **Supplementary Table 3**. The classification accuracy was no longer improved by increasing the panel size that incorporated additional clinical covariates, including histology, tumor stage, lymph node stage, metastasis stage, *etc.* (**Figure S8C**).

Intriguingly, the predicted immune-activated MPMs showed a better prognosis than the predicted non-immune MPMs (**Figure 5E**), demonstrating that the 12-gene classifier is a good predictor of survival. Multivariate Cox regression analysis confirmed that the 12-gene classifier was a promising and independent biomarker set for predicting patient survival (**Figure S9A**). To assess the expression patterns of 12 genes, we correlated their expression levels with immune cell infiltrates estimated by CIBERSORT. Hence, two gene categories were identified with distinct expression patterns: genes within category one (*GZMA, APOBEC3G, BTN3A2, TRAT1, HCK, BIN2*, and *SLAMF8*) showed a positive relationship with the abundance of various types of T cells, while category two (*TNC, TNFAIP6, HAS2, SH2B3*, and *ITGA4*) was associated with myeloid components (**Figure S10A**).

## Discussion

The recently published result of Checkmate743 has established the position of dual-target immunotherapy in first-line treatment for MPMs (6). This new therapy pattern raises demands for predicting patients capable of benefiting from ICIs. TMB and PD-L1 are two well-recognized biomarkers for predicting the efficiency of immunotherapy with a wide application (38, 39). Although KEYNOTE-158 has confirmed the clinical efficacy of Keytrude in tumors harboring a TMB≥10 across multiple solid tumors including mesothelioma (40), our analyses revealed a deficiency of TMB with an average expression around 0.5 (**Figure S5D**). By contrast, expression of PD-L1 ranged from 22 to 42% in MPM patients with a variety of assessment methods (41–43). High PD-L1 expression seemed to be correlated with adverse clinical outcomes for MPMs (43). However, the optimal cutoff score used for predicting prognosis or ICI response remains to be determined. Moreover, there is no consensus regarding PD-L1’s predictive value in recognizing potential beneficial patients upon immunotherapy. As a single biomarker, TMB or PD-L1 is insufficient to cover all the intrinsic and environmental factors driving immune heterogeneity of MPMs, and thus has its limitation in being applied to clinical practice.

From this, we speculate that proposing a subtyping system for MPM can promote our understanding of TME heterogeneity and is critical for improving the efficacy of current immunotherapeutic strategies. Previously, classical classification patterns were defined to stratify the immune microenvironment of solid tumors into four types based on the presence or absence of tumor-infiltrating lymphocytes (TILs) and PD-L1 expression, including Type I cancers (PD-L1^+^ TILs^+^), Type II cancers (PD-L1^−^ TILs^−^), Type III cancers (PD-L1^+^ TILs^−^), Type IV cancers (PD-L1^−^TILs^+^) (44). Correlating this stratification system with our classification identified immune-activated MPMs as Type I cancers, which were more likely to benefit from anti-PD1/anti-PD-L1 therapy. Similar to previous findings (45), PD-L1 showed greater expression in immune-activated MPMs accompanied by infiltration of cytotoxic T-cells (**Figure S4A**). For the explanations, the persistent involvement of T cells in tumor immunity was balanced by PD-L1 engagement, which is induced by IFN-γ as an adaptive mechanism and thus exactly appropriate for anti-PD1/PD-L1 therapy (46).

Meanwhile, we observed patients within immune-suppressed MPMs contained substantial myeloid components (MDSCs, TAMs) and Tregs responsible for mediating immune tolerance. To take control of this predicted Type IV cancers-associated MPMs, we deemed that simple using combination regiments containing antibodies against PD-L1, CTLA-4, and other immune checkpoints may not be enough considering the immune-suppressive status. TAMs can suppress T cell activity *via* upregulating checkpoint molecules, indirectly crosstalk with Tregs, and secreting immunosuppressive cytokines (47), which eventually results in ICI treatment failure. Thus, immune-suppressed MPMs may benefit from inhibiting the CSF1/CSF1R pathway, a key participant in the proliferation, differentiation, and recruitment of macrophages (48). The efficacy of the CSF1/CSF1R antibody, Cabiralizumab, combined with nivolumab in advanced solid tumors, is currently being investigated in a phase 2 trial (NCT02526017).

The remaining non-immune MPMs accounted for a large part and held the characteristics of Type II (PD-L1 negative with no TIL indicating immune ignorance) or Type III cancers (PD-L1 positive with no TIL indicating intrinsic induction). For these “cold” tumors, enhancing the immunogenicity of tumor cells to attract more T cell infiltration should be prioritized. To achieve it, developing therapies to induce exposure to tumor antigens would be a primary measure to take. As an ideal way to cause immunogenic cell death and liberate neo-antigens, radiotherapy has been combined with immunotherapy to enhance CD8 T-cell responses (49). For the consideration of inducing vascular normalization, anti-angiogenic therapy allows more TILs to access the TME and thus improves the efficiency of ICI through augmenting immune recognition (50). Some treatment guidelines recommended the addition of the anti-angiogenic agent bevacizumab to platinum plus pemetrexed chemotherapy as first-line treatment for selected MPM patients (51, 52). Given the durable survival benefit seen in CheckMate 743, combining nivolumab plus ipilimumab with other therapies, including anti-angiogenic agents, merits further investigation to determine whether tumor response can be enhanced.

Clinical survival is one of our primary concerns for this classification scheme. Our work identified that immune-activated MPM patients exhibited more favourable prognoses relative to immune-suppressed MPM patients in both two cohorts, while conflicting data exist regarding the patient survival of non-immune MPMs. Those seemingly contradictory data might be attributed to different sample properties, including TMN staging, histology, and sample size. For the non-immune MPMs, delineating molecular features using the NMF algorithm (**Figure 1A**) has summarized three distinct functional modules/subclasses: Cell cycle, Epithelial/IFN response, and ECM subtypes. Further work should investigate these heterogeneous molecular patterns and their associations with immune reprogramming and clinical outcome.

The limitation of the current study is the lack of histology evaluation for each sample. In particular, all the analyses were solely based on bulk transcriptome and cell type deconvolution. The recent finding suggested that some SCLC (small cell lung cancer) cases do contain not low immune cells that were more immunological sequestrated (53). A tumor-immune microenvironment is well-organized and structured from compartmentalized to mixed patterns relating to survival (54). With the advances in the spatial transcriptome, future work should pay more attention to the spatial distribution of immune cells in MPMs, which can help choose appropriate patients to receive the immunotherapy. Besides, the ability of our immune subtypes to predict responses to different immunotherapeutic approaches is worth exploring in clinical trials or real-world studies.

To sum up, we developed a novel and feasible subtype classification system for delineating MPM immune features. We demonstrate that this classification system can be exploited to guide immunotherapy strategies, providing critical biological insights into the mechanisms driving tumor heterogeneity. A machine learning-based 12-gene classifier was exploited to simplify classified procedures, holding promise in clinical translation and prognostic determination.

## Data availability statement

The original contributions presented in the study are included in the article/**Supplementary Material**. Further inquiries can be directed to the corresponding authors.

## Author contributions

KY was associated with conceptualization, methodology design, data validation, visualization, writing an original draft, and supervision of the study. TXY and TY conducted data collection and curation. YY took charge of formal analysis. FL performed the investigation, project administration, and conducted writing review & editing. All authors contributed to the article and approved the submitted version.

## Acknowledgments

All authors would like to thank the patients and investigators who participated in TCGA and GEO for providing data.

## Conflict of interest

The authors declare that the research was conducted in the absence of any commercial or financial relationships that could be construed as a potential conflict of interest.

## Publisher’s note

All claims expressed in this article are solely those of the authors and do not necessarily represent those of their affiliated organizations, or those of the publisher, the editors and the reviewers. Any product that may be evaluated in this article, or claim that may be made by its manufacturer, is not guaranteed or endorsed by the publisher.
