# Supplementary material for: Unraveling tumor microenvironment heterogeneity in malignant pleural mesothelioma identifies biologically distinct immune subtypes enabling prognosis determination
Source: Front Oncol. 2022 Sep 27;12:995651. doi: 10.3389/fonc.2022.995651 (PMC9552848; doi:10.3389/fonc.2022.995651)
Supplement: Supplementary file 1 [file DataSheet_1.docx]

Supplementary Material

Unraveling Tumor Microenvironment Heterogeneity in Malignant Pleural Mesothelioma Identifies Biologically Distinct Immune Subtypes Enabling Prognosis Determination

Kaidi Yang^1,*^, Tongxin Yang^1^, Tao Yang^1^, Ye Yuan^2^, Fang Li^1,*^

^1^Department of Oncology, Hainan Hospital of Chinese People's Liberation Army General Hospital, Sanya, Hainan Province, PR China.

^2^Institute of Pathology and Southwest Cancer Center, Southwest Hospital, Third Military Medical University (Army Medical University), Chongqing, P. R. China.

*** Correspondence:**Kaidi Yang and Fang Li,

Department of Oncology, Hainan Hospital of Chinese People's Liberation Army General Hospital, Sanya 400038, China.

E-mail: 13601261747@139.com (F, Li) and lampirl@163.com (K. Yang)


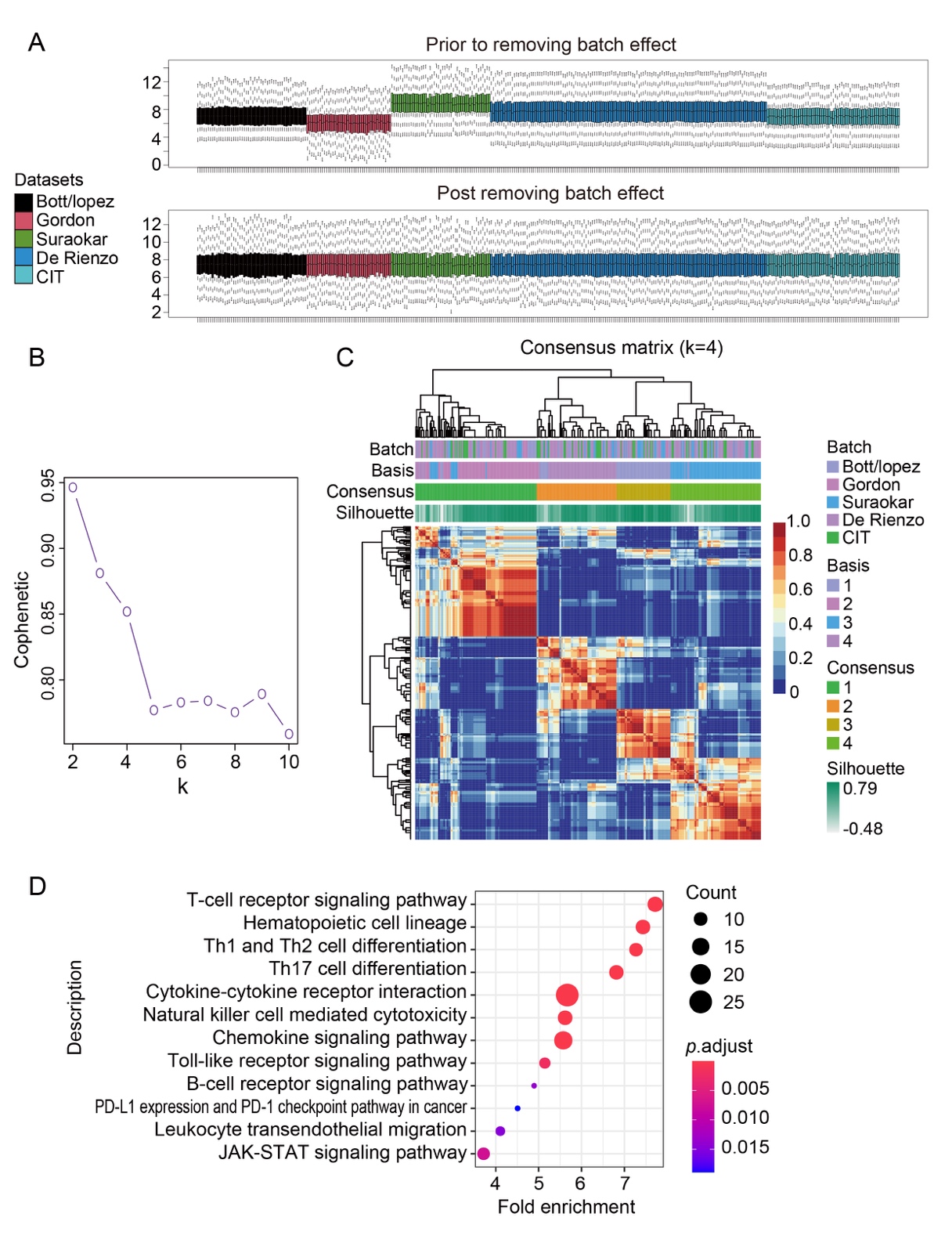


**Figure S1.** Related to **Figure 1**.

**(A)** The two boxplots showing the deviation of mean gene expression prior to- and post-removal of batch effect across the five mesothelioma datasets using the *Combat* function. **(B)** Cophenetic correlation plot for k = 2 to k = 10 classes after NMF for 333 malignant pleural mesothelioma (MPM) samples. **(C)** The consensus matrice for k = 4 is shown. **(D)** KEGG enrichment analyses of top 200 weighted genes in the immune module. The index of x-axis indicates the fold enrichment of each term as defined by the ratio of GeneRatio/BgRatio as defined by *ClusterProfiler* package. The bubble size indicates the number of genes in each term, and different colors correspond to different adjusted *p*-values. The *p*-values are adjusted by the Benjamini-Hochberg method.


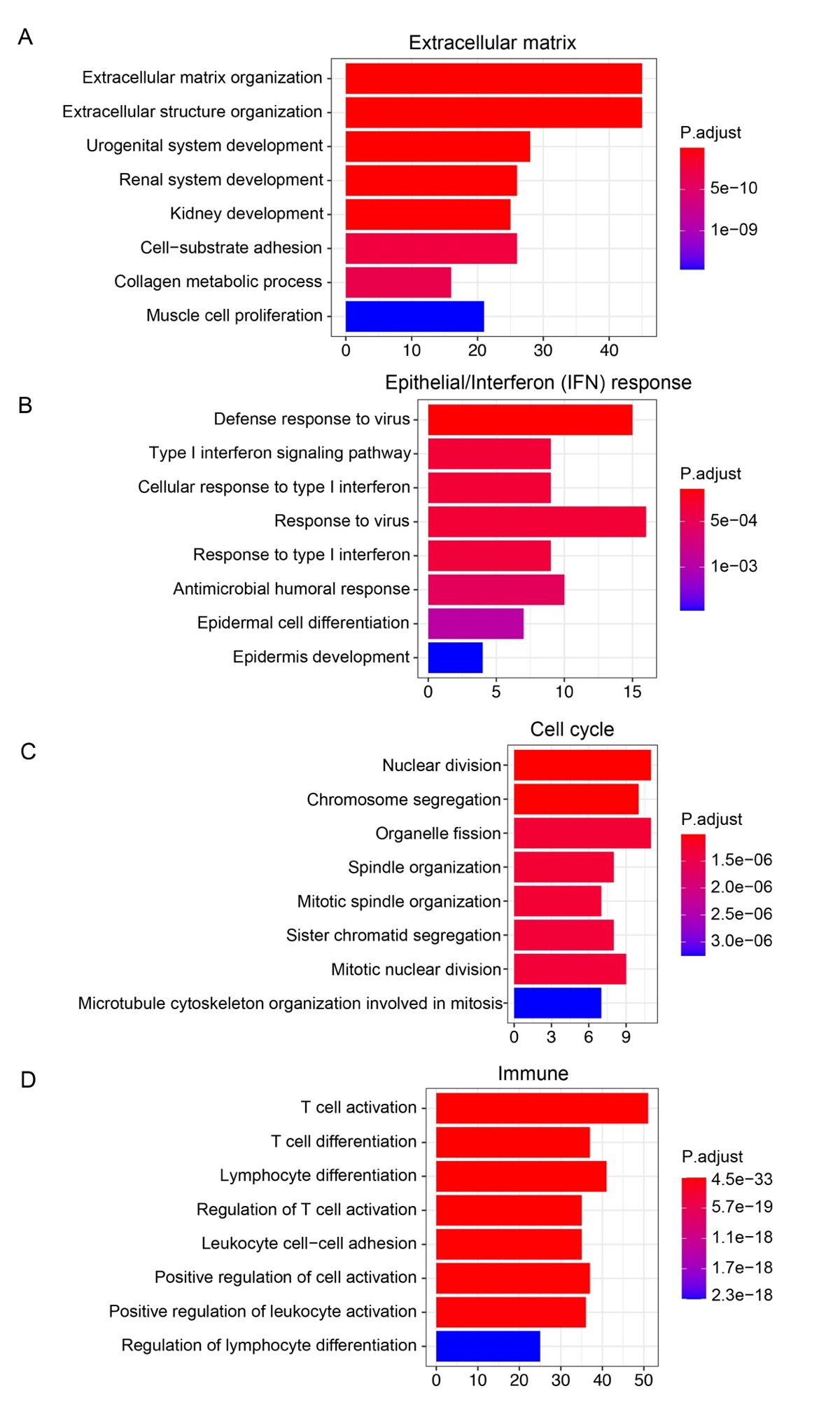


**Figure S2.** Identification of characteristics of each NMF module via over-representation (ORA) analysis. Related to **Figure 1**.

(**A-D**) Bar plots showing the results of ORA analysis for the top weighted genes of each NMF module. The index in the x-axis indicates the number of genes in each term and various colors correspond to different adjusted *P*-values, which are adjusted by the Benjamini-Hochberg method.


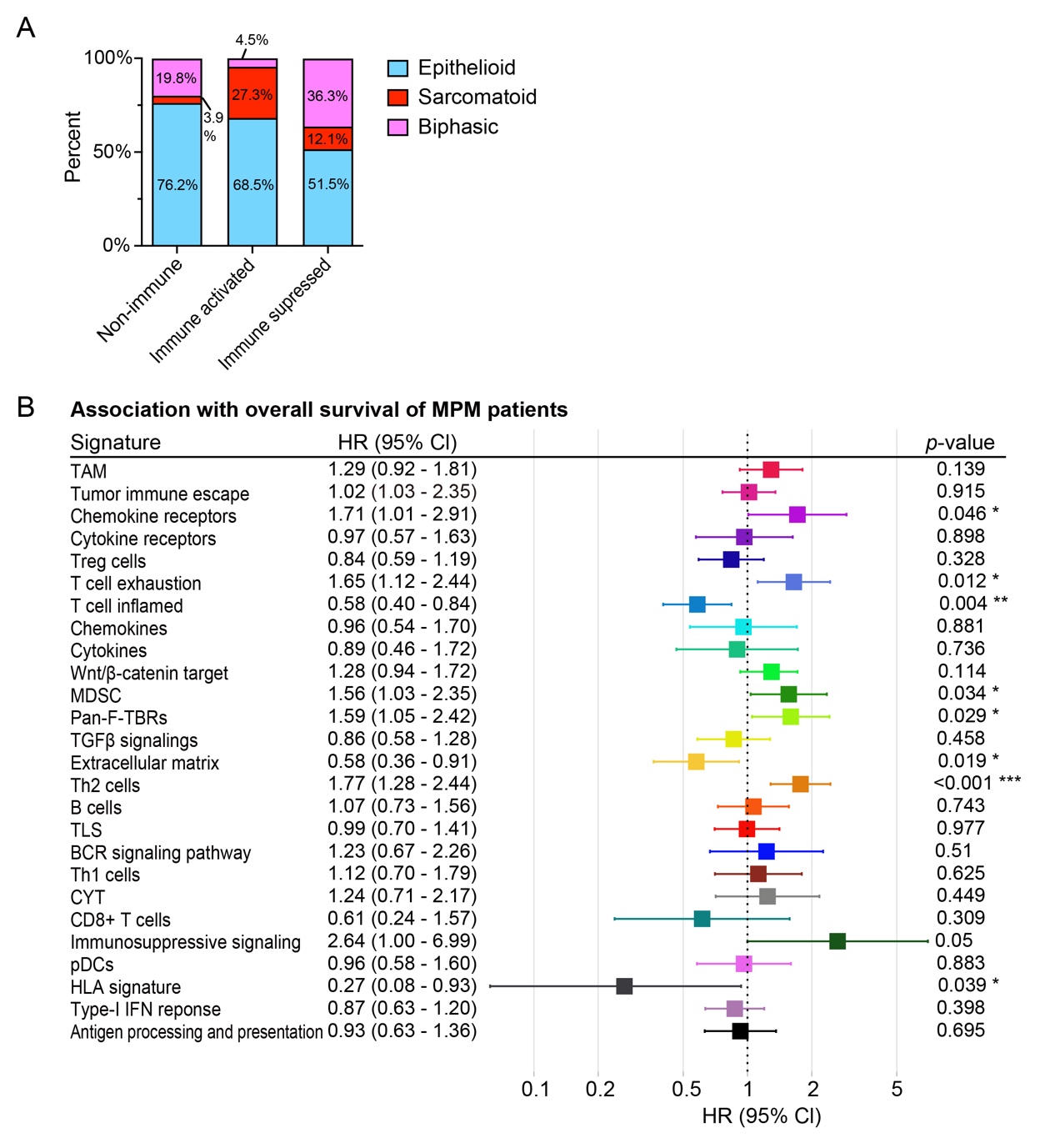


**Figure S3.** Related to **Figure 2**.

**(A)** Percentage column plots show the distribution of MPM histological subtypes across the three immune subtypes of batch correlated three MPM cohorts. **(B)** Forest plot shows the effect of different immune-related signatures on overall survival using multivariate Cox regression analysis. The x-axis represents the hazard ratio with the reference line (dashed), hazard ratios (quadrate), and 95% confidence interval (whiskers). HR, Hazard ratio; CL, Confidence level; TAM, Tumor-associated macrophage; MDSC, Myeloid-derived suppressor cell; TLS, Tertiary lymphoid structure; CYT, Cytolytic activity. pDCs, Plasmacytoid dendritic cell; Pan-F-TBRs, Pan fibroblast TGF-β response signature.


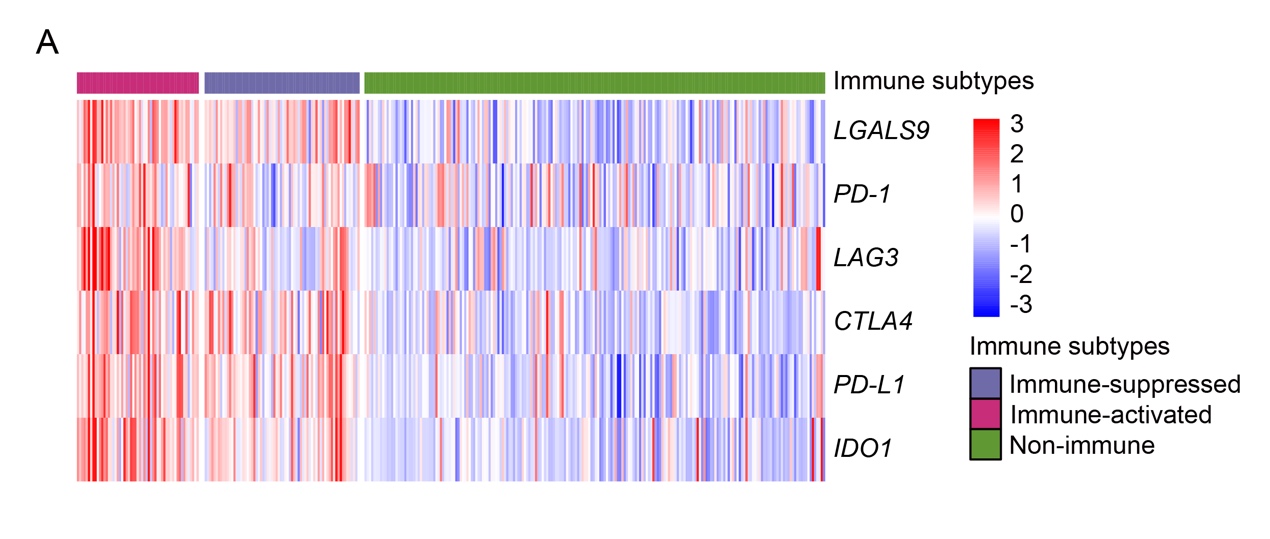


**Figure S4.** Related to **Figure 2**.

**(A)** Heatmap illustrating the expression levels of six immune checkpoint-related genes in the different immune subtypes of MPMs.


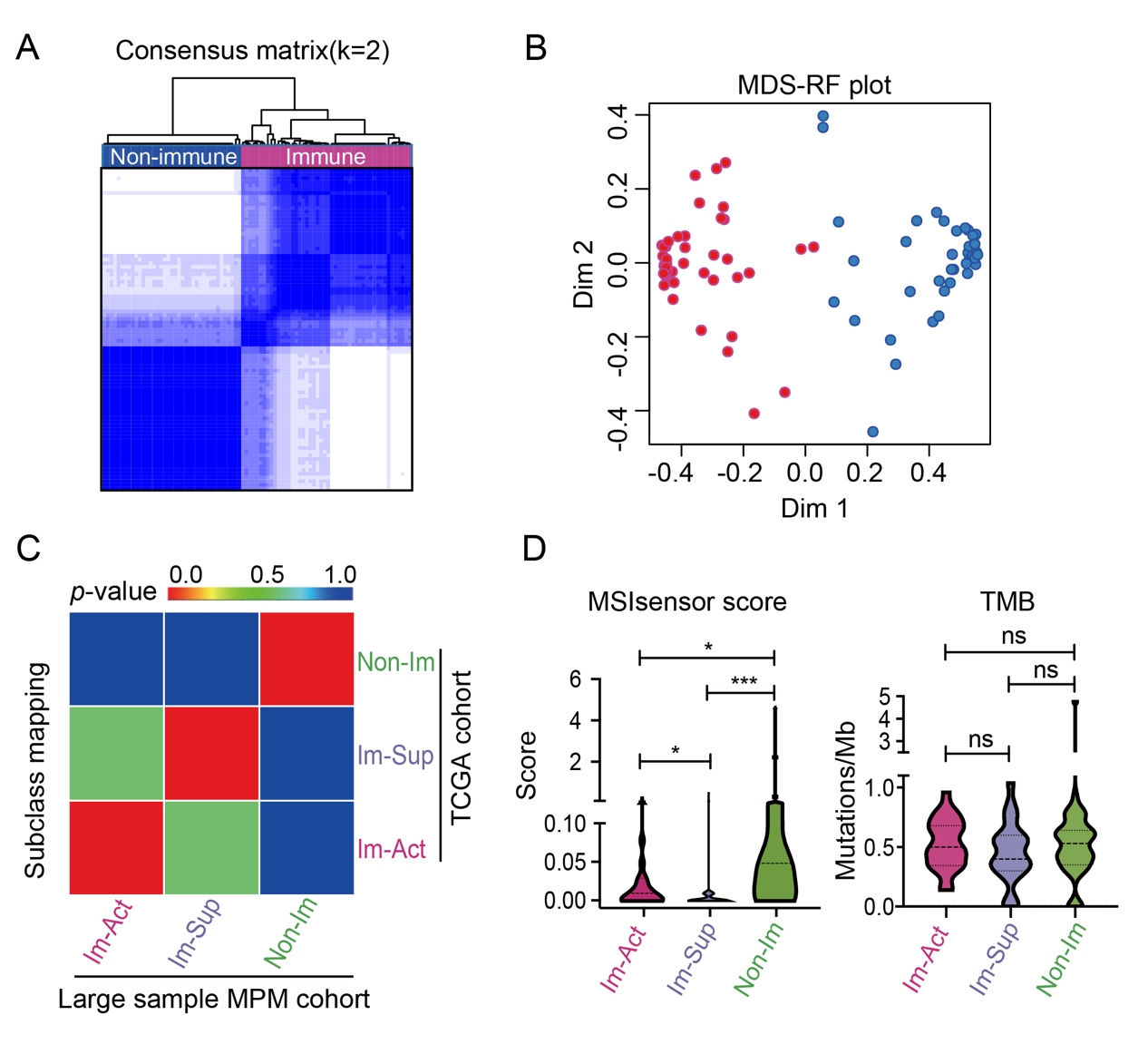


**Figure S5.** Related to **Figure 3**.

**(A)** Consensus clustering based on the top 200 exemplar genes expression identified two subgroups for the TCGA-MESO cohort. **(B)** The multidimensional scaling random forest (MDS-RF) refined the classification and divided TCGA-MESO samples into immune and non-immune subtypes. **(C)** Subclass mapping analysis of the three subgroups using the TCGA-MESO and combined large-sample MPM datasets. The color labeled in each cell reflects the significance of each subclass association. Im-Act, Immune-activated; Im-Sup, Immune-suppressed. **(D)** Values of microsatellite instability (MSI) score and tumor mutation burden (TMB) across different immune subtypes.


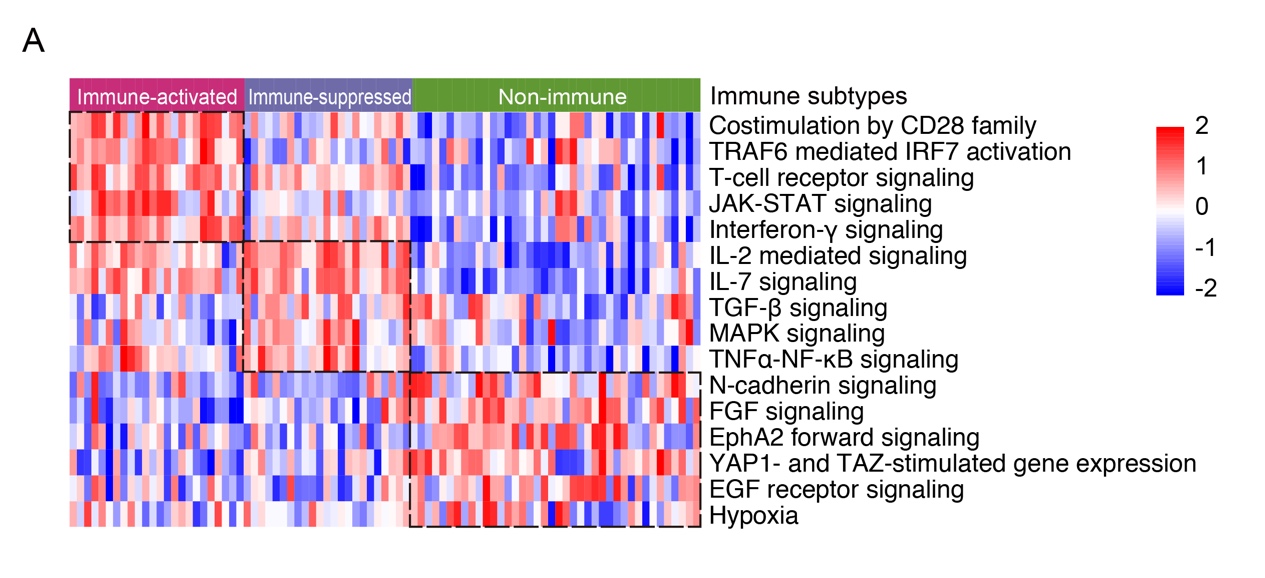


**Figure S6.** Related to **Figure 3**.

**(A)** Heatmap illustrating the expression levels of representative signaling pathways for the three immune subtypes.


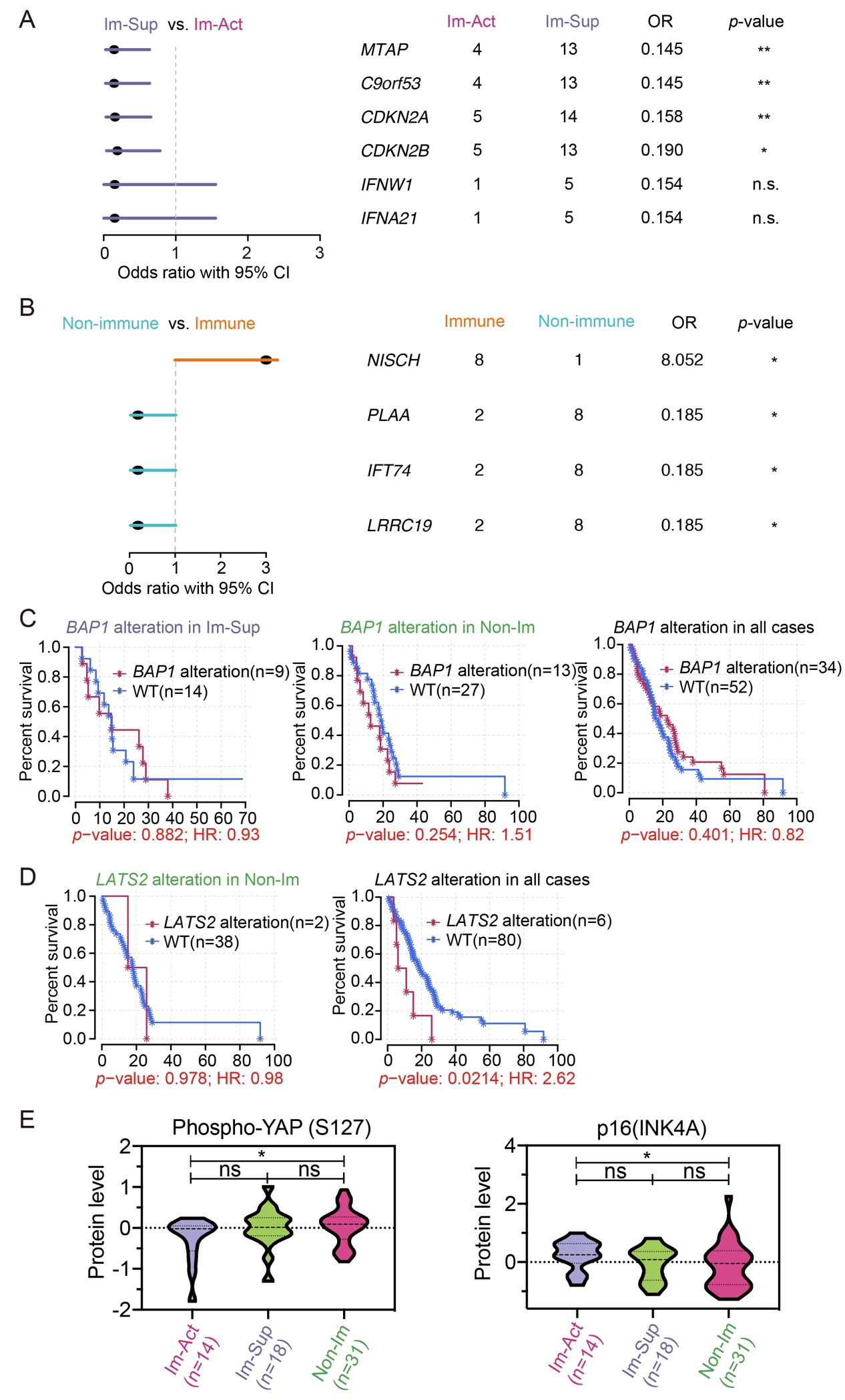


**Figure S7.** Related to **Figure 4**.

**(A-B)** Subtype-based pairwise comparisons of genomic alteration rates through forestplots, genes significantly enriched through comparisons were presented. OR, Odd ratio; **p < 0.01; *p < 0.05; n.s., Not significant. **(C-D)** Kaplan-Meier curves of the overall survival in the corresponding subtype of MPM patients stratified by *BAP1* (C) and *LATS2* (D) genomic alteration status. WT, Wild-type. **(E)** Protein level of YAP phosphorylation (S127) and p16(INK4A) for MPM samples with different immune subtypes. *p < 0.05; ns, Not significant.


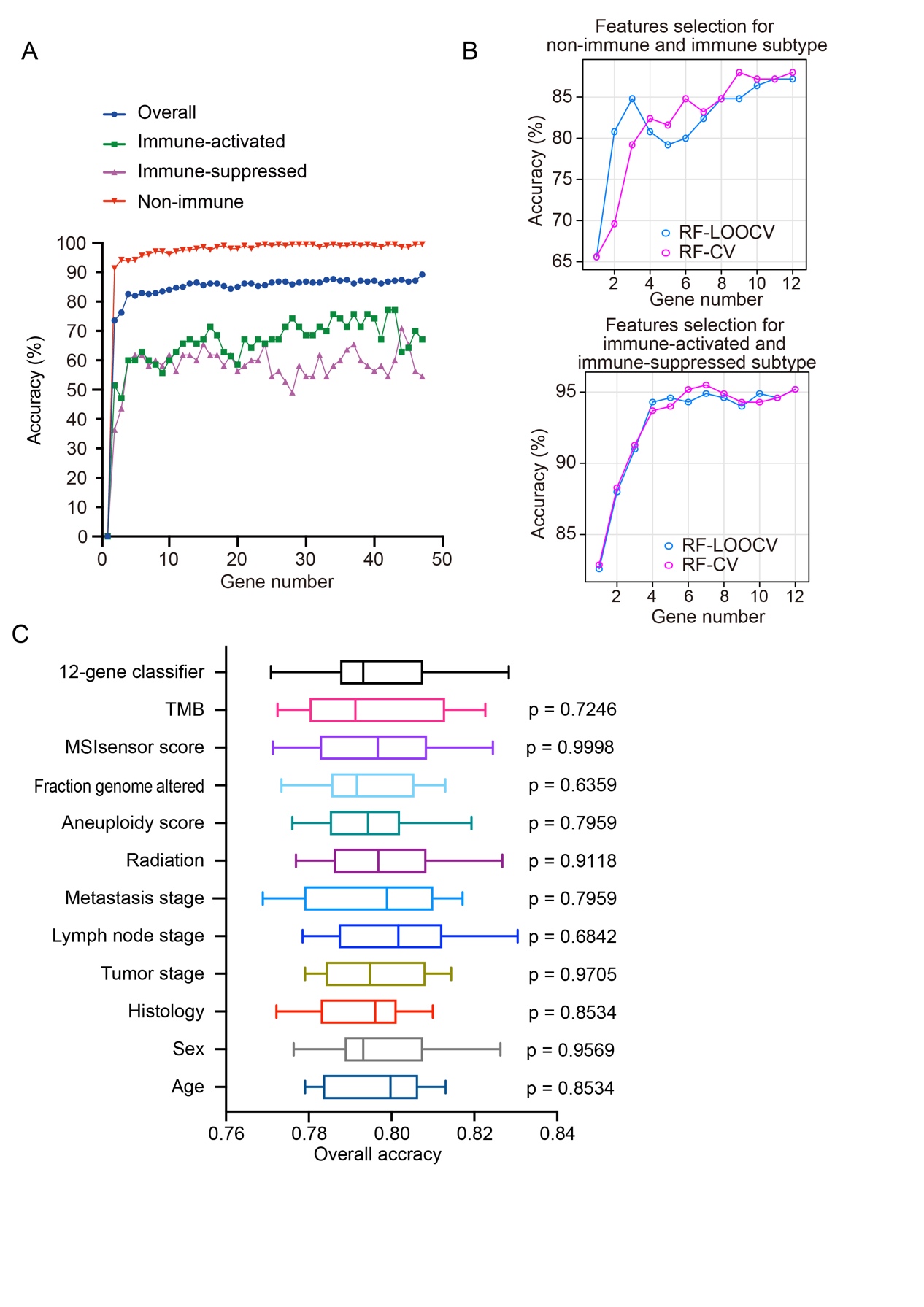


**Figure S8.** Related to **Figure 5**.

**(A)** Dot and line charts illustrating recursive processes of random forest (RF) with Leave-one-out cross-validation (LOOCV) for predicting the whole cohort and each immune subtype. The four variation trends of accuracy were presented with the increasing sizes of panel, in which features are ranked and added in sequence by predictive power. **(B)** Line graphs illustrating variation trend of accuracy computed by RF-LOOCV and RF-CV algorithms with stepwise recursive feature elimination (RFE) process. The x-axis suggested a different number of variable combinations. **(C)** Impact of clinical covariates on the classification accuracy of 12-gene classifier when added to the random forest model.


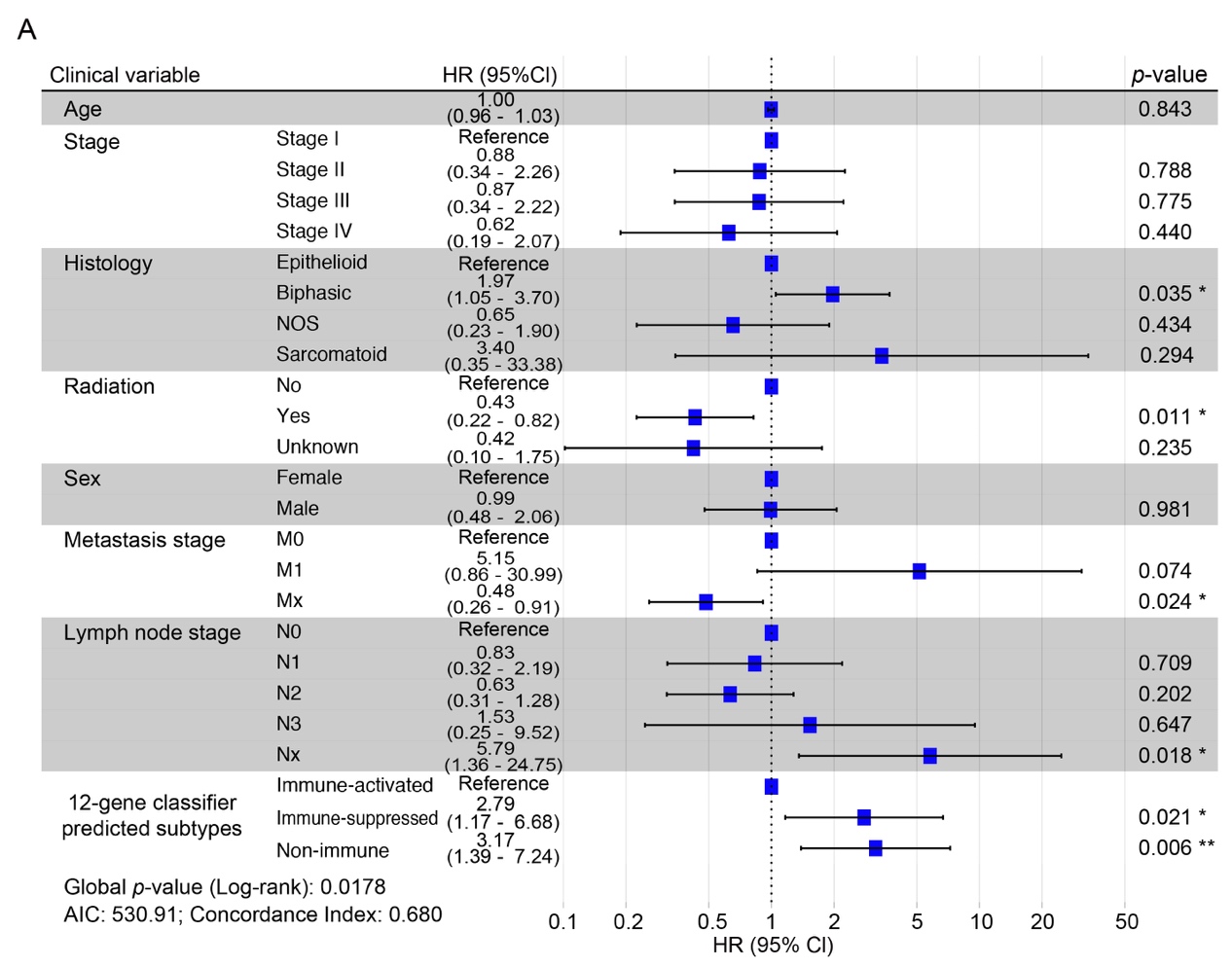
**Figure S9.** Related to **Figure 5**.

**(A)** Forest plot showing the effect of different clinical variables on overall survival using multivariate Cox regression analysis. The x-axis represents the hazard ratio with the reference line (dashed), hazard ratios (blue quadrate), and 95% confidence interval (whiskers).


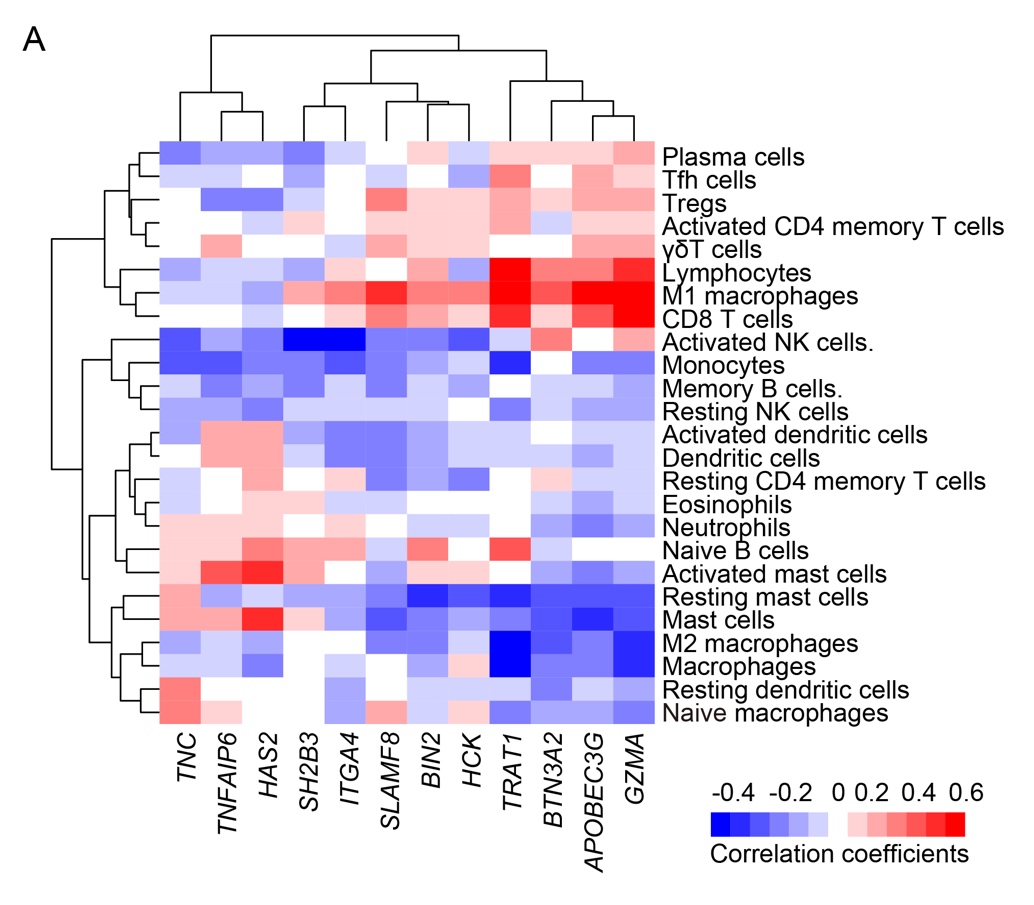


**Figure S10.** Related to **Figure 5**.

**(A)** Heatmap illustrating Pearson correlation coefficients between 12-gene classifier and CIBERSORT estimates of immune cell infiltrates.
