# Supplementary material for: Unraveling tumor microenvironment heterogeneity in malignant pleural mesothelioma identifies biologically distinct immune subtypes enabling prognosis determination
Source: Front Oncol. 2022 Sep 27;12:995651. doi: 10.3389/fonc.2022.995651 (PMC9552848; doi:10.3389/fonc.2022.995651)
Supplement: Supplementary file 2 [file Table_1.docx]

| **Table S1. Summary of the information of enrolled malignant pleural mesothelial cohorts** | | | | | |
| --- | --- | --- | --- | --- | --- |
| Dataset | Author/Group | Data array | Number of MPM patients | Number of qualified samples | Reference |
| TCGA-MESO | TCGA | RNA sequencing | 87 | 87 | https://xenabrowser.net/ |
| MSKCC-GSE29354 | Bott/lopez | Affymetrix Human Genome U133A Array | 53 | 52 | https://www.ncbi.nlm.nih.gov/geo/query/acc.cgi?acc=GSE29354 |
| GSE2549 | Gordon | Affymetrix Human Genome U133A Array | 40 | 40 | <https://www.ncbi.nlm.nih.gov/geo/query/acc.cgi?acc=GSE2549> |
| GSE51024 | Suraokar | Affymetrix Human Genome U133 Plus 2.0 Array | 55 | 47 | <https://www.ncbi.nlm.nih.gov/geo/query/acc.cgi?acc=GSE51024> |
| GSE163722 | De Rienzo | Affymetrix Human Gene 1.1 ST Array [transcript (gene) version] | 131 | 131 | <https://www.ncbi.nlm.nih.gov/geo/query/acc.cgi?acc=GSE163722> |
| E-MTAB-6877 | CIT | Affymetrix Human Gene 2.0 ST Array [transcript (gene) version] | 63 | 63 | https://www.ebi.ac.uk/arrayexpress/experiments/E-MTAB-6877/ |
