# Supplementary material for: Unraveling tumor microenvironment heterogeneity in malignant pleural mesothelioma identifies biologically distinct immune subtypes enabling prognosis determination
Source: Front Oncol. 2022 Sep 27;12:995651. doi: 10.3389/fonc.2022.995651 (PMC9552848; doi:10.3389/fonc.2022.995651)
Supplement: Supplementary file 5 [file Table_4.docx]

| **Table S4. The classification results with different methods for 333 MPM patients, related to Figure 1D and Figure 2A.** | | | | | | | | |
| --- | --- | --- | --- | --- | --- | --- | --- | --- |
| **Sample ID** | **Batches** | **Consensus clusters** | **NMF modules** | **Immune subtypes k2** | **Immune module weight** | **Immune module score** | **Immune subtypes k3** | **ICI response** |
| GSM4984443_MCER83.CEL | GSE163722 | Immune cluster | Immune | Immune | 0.297 | 1611.183 | Immune-suppressed | Non-responder |
| GSM4984393_MCER26.CEL | GSE163722 | Immune cluster | Immune | Immune | 0.263 | 1630.264 | Immune-suppressed | Non-responder |
| GSM4984442_MCER82.CEL | GSE163722 | Immune cluster | ECM | Immune | 0.243 | 1866.825 | Immune-suppressed | Non-responder |
| GSM4984458_MCER105.CEL | GSE163722 | Immune cluster | Immune | Immune | 0.271 | 1841.335 | Immune-suppressed | Responder |
| GSM49566 | GSE2549 | Immune cluster | Immune | Immune | 0.269 | 1628.277 | Immune-suppressed | Non-responder |
| GSM725587.CEL | MSKCC-GSE29354 | Immune cluster | Immune | Immune | 0.285 | 2014.851 | Immune-suppressed | Responder |
| GSM4984407_MCER42_HuGene-1_1-st-v1_F05.CEL | GSE163722 | Immune cluster | ECM | Immune | 0.212 | 1677.479 | Immune-suppressed | Non-responder |
| GSM1235021 | GSE51024 | Immune cluster | ECM | Immune | 0.233 | 1500.730 | Immune-suppressed | Non-responder |
| GSM4984496_MCER148_HuGene-1_1-st-v1_G08.CEL | GSE163722 | Immune cluster | ECM | Immune | 0.207 | 1196.472 | Immune-suppressed | Non-responder |
| GSM1235037 | GSE51024 | Immune cluster | Immune | Immune | 0.290 | 2039.923 | Immune-suppressed | Non-responder |
| DJN_110_HuGene2.CEL | E-MTAB-6877 | Immune cluster | ECM | Immune | 0.246 | 1770.345 | Immune-suppressed | Non-responder |
| GSM49597 | GSE2549 | Immune cluster | ECM | Immune | 0.249 | 1575.848 | Immune-suppressed | Non-responder |
| GSM725569.CEL | MSKCC-GSE29354 | Immune cluster | ECM | Immune | 0.219 | 1461.039 | Immune-suppressed | Responder |
| GSM4984388_MCER20.CEL | GSE163722 | Immune cluster | Immune | Immune | 0.285 | 2191.143 | Immune-suppressed | Responder |
| GSM4984429_MCER67.CEL | GSE163722 | Immune cluster | Immune | Immune | 0.252 | 1413.686 | Immune-suppressed | Non-responder |
| GSM725615.CEL | MSKCC-GSE29354 | Non-immune cluster | ECM | Immune | 0.214 | 1495.455 | Immune-suppressed | Non-responder |
| GSM4984467_MCER115.CEL | GSE163722 | Immune cluster | Immune | Immune | 0.234 | 1420.417 | Immune-suppressed | Non-responder |
| GSM4984490_MCER142.CEL | GSE163722 | Immune cluster | ECM | Immune | 0.207 | 1846.279 | Immune-suppressed | Non-responder |
| DJN_102_HuGene2.CEL | E-MTAB-6877 | Immune cluster | ECM | Immune | 0.258 | 2176.762 | Immune-suppressed | Non-responder |
| GSM4984425_MCER63.CEL | GSE163722 | Immune cluster | Epithelial/IFN response | Immune | 0.237 | 2124.344 | Immune-suppressed | Responder |
| GSM1235095 | GSE51024 | Immune cluster | ECM | Immune | 0.249 | 2260.859 | Immune-suppressed | Non-responder |
| GSM4984459_MCER106.CEL | GSE163722 | Immune cluster | ECM | Immune | 0.135 | 1519.562 | Immune-suppressed | Non-responder |
| DJN_115_HuGene2.CEL | E-MTAB-6877 | Immune cluster | Immune | Immune | 0.325 | 1422.088 | Immune-suppressed | Non-responder |
| GSM725608.CEL | MSKCC-GSE29354 | Non-immune cluster | Cell cycle | Immune | 0.227 | 1345.624 | Immune-suppressed | Non-responder |
| GSM49594 | GSE2549 | Immune cluster | ECM | Immune | 0.244 | 1837.064 | Immune-suppressed | Responder |
| GSM4984471_MCER121.CEL | GSE163722 | Immune cluster | Immune | Immune | 0.264 | 1708.902 | Immune-suppressed | Non-responder |
| GSM725594.CEL | MSKCC-GSE29354 | Immune cluster | Immune | Immune | 0.257 | 1603.889 | Immune-suppressed | Non-responder |
| GSM4984401_MCER35.CEL | GSE163722 | Immune cluster | ECM | Immune | 0.177 | 1434.687 | Immune-suppressed | Non-responder |
| GSM4984436_MCER75.CEL | GSE163722 | Immune cluster | ECM | Immune | 0.217 | 1948.662 | Immune-suppressed | Non-responder |
| GSM4984456_MCER101.CEL | GSE163722 | Immune cluster | Epithelial/IFN response | Immune | 0.165 | 1515.986 | Immune-suppressed | Non-responder |
| GSM49601 | GSE2549 | Immune cluster | Immune | Immune | 0.233 | 1986.225 | Immune-suppressed | Responder |
| DJN_092_HuGene2.CEL | E-MTAB-6877 | Immune cluster | Immune | Immune | 0.301 | 1590.862 | Immune-suppressed | Non-responder |
| GSM725595.CEL | MSKCC-GSE29354 | Immune cluster | Epithelial/IFN response | Immune | 0.217 | 1890.766 | Immune-suppressed | Responder |
| DJN_123_HuGene2.CEL | E-MTAB-6877 | Immune cluster | Immune | Immune | 0.252 | 1582.488 | Immune-suppressed | Non-responder |
| GSM1235089 | GSE51024 | Immune cluster | ECM | Immune | 0.179 | 1591.375 | Immune-suppressed | Responder |
| GSM1235087 | GSE51024 | Immune cluster | ECM | Immune | 0.200 | 1630.161 | Immune-suppressed | Responder |
| GSM4984431_MCER69_HuGene-1_1-st-v1_G01.CEL | GSE163722 | Immune cluster | ECM | Immune | 0.196 | 1421.226 | Immune-suppressed | Non-responder |
| DJN_118_HuGene2.CEL | E-MTAB-6877 | Immune cluster | Immune | Immune | 0.329 | 2300.831 | Immune-suppressed | Responder |
| GSM4984463_MCER110.CEL | GSE163722 | Non-immune cluster | Immune | Immune | 0.282 | 1463.388 | Immune-suppressed | Non-responder |
| GSM4984430_MCER68.CEL | GSE163722 | Immune cluster | Immune | Immune | 0.341 | 2218.950 | Immune-suppressed | Non-responder |
| GSM725606.CEL | MSKCC-GSE29354 | Immune cluster | Immune | Immune | 0.259 | 1786.637 | Immune-suppressed | Non-responder |
| GSM4984414_MCER50.CEL | GSE163722 | Immune cluster | Immune | Immune | 0.238 | 1547.412 | Immune-suppressed | Responder |
| GSM4984470_MCER120.CEL | GSE163722 | Non-immune cluster | Epithelial/IFN response | Immune | 0.232 | 1593.419 | Immune-suppressed | Responder |
| GSM49602 | GSE2549 | Immune cluster | Immune | Immune | 0.232 | 1539.320 | Immune-suppressed | Non-responder |
| DJN_105_HuGene2.CEL | E-MTAB-6877 | Immune cluster | Immune | Immune | 0.300 | 1509.856 | Immune-suppressed | Non-responder |
| GSM49595 | GSE2549 | Immune cluster | ECM | Immune | 0.215 | 1355.498 | Immune-suppressed | Non-responder |
| DJN_014_HuGene2.CEL | E-MTAB-6877 | Immune cluster | Immune | Immune | 0.340 | 1433.655 | Immune-suppressed | Non-responder |
| GSM49583 | GSE2549 | Immune cluster | ECM | Immune | 0.236 | 2082.530 | Immune-suppressed | Non-responder |
| GSM4984396_MCER30_HuGene-1_1-st-v1_H11.CEL | GSE163722 | Immune cluster | Cell cycle | Immune | 0.273 | 1261.123 | Immune-suppressed | Non-responder |
| GSM49565 | GSE2549 | Immune cluster | ECM | Immune | 0.217 | 1679.604 | Immune-suppressed | Responder |
| GSM1235023 | GSE51024 | Immune cluster | ECM | Immune | 0.259 | 2349.223 | Immune-suppressed | Responder |
| GSM4984475_MCER126.CEL | GSE163722 | Immune cluster | Immune | Immune | 0.345 | 1738.891 | Immune-suppressed | Non-responder |
| GSM49578 | GSE2549 | Immune cluster | Epithelial/IFN response | Immune | 0.220 | 1534.933 | Immune-suppressed | Responder |
| GSM49570 | GSE2549 | Immune cluster | Immune | Immune | 0.298 | 1817.225 | Immune-suppressed | Non-responder |
| GSM725591.CEL | MSKCC-GSE29354 | Immune cluster | Epithelial/IFN response | Immune | 0.221 | 1757.864 | Immune-suppressed | Responder |
| DJN_083_HuGene2.CEL | E-MTAB-6877 | Immune cluster | Epithelial/IFN response | Immune | 0.225 | 2327.876 | Immune-suppressed | Non-responder |
| GSM4984495_MCER147_HuGene-1_1-st-v1_G07.CEL | GSE163722 | Immune cluster | Immune | Immune | 0.324 | 1639.784 | Immune-suppressed | Non-responder |
| GSM4984447_MCER87.CEL | GSE163722 | Immune cluster | ECM | Immune | 0.244 | 2615.835 | Immune-suppressed | Non-responder |
| DJN_119_HuGene2.CEL | E-MTAB-6877 | Immune cluster | Cell cycle | Immune | 0.251 | 1420.136 | Immune-suppressed | Responder |
| GSM725617.CEL | MSKCC-GSE29354 | Immune cluster | Immune | Immune | 0.387 | 2666.894 | Immune-suppressed | Non-responder |
| GSM4984412_MCER48.CEL | GSE163722 | Immune cluster | Immune | Immune | 0.272 | 1923.127 | Immune-suppressed | Responder |
| GSM49589 | GSE2549 | Immune cluster | Immune | Immune | 0.355 | 2402.377 | Immune-suppressed | Non-responder |
| GSM4984460_MCER107.CEL | GSE163722 | Immune cluster | ECM | Immune | 0.260 | 2425.578 | Immune-suppressed | Non-responder |
| GSM4984385_MCER16_HuGene-1_1-st-v1_H05.CEL | GSE163722 | Immune cluster | ECM | Immune | 0.148 | 1938.192 | Immune-suppressed | Non-responder |
| GSM725600.CEL | MSKCC-GSE29354 | Immune cluster | ECM | Immune | 0.243 | 2528.282 | Immune-suppressed | Non-responder |
| DJN_099_HuGene2.CEL | E-MTAB-6877 | Immune cluster | ECM | Immune | 0.263 | 2395.913 | Immune-suppressed | Non-responder |
| GSM4984484_MCER136_HuGene-1_1-st-v1_F11.CEL | GSE163722 | Immune cluster | Immune | Immune | 0.244 | 1894.830 | Immune-suppressed | Responder |
| DJN_087_HuGene2.CEL | E-MTAB-6877 | Immune cluster | Cell cycle | Immune | 0.275 | 1256.799 | Immune-suppressed | Non-responder |
| GSM4984438_MCER79_HuGene-1_1-st-v1_G04.CEL | GSE163722 | Immune cluster | Epithelial/IFN response | Immune | 0.188 | 1807.268 | Immune-suppressed | Responder |
| DJN_122_HuGene2.CEL | E-MTAB-6877 | Immune cluster | Immune | Immune | 0.258 | 1703.043 | Immune-suppressed | Responder |
| GSM4984411_MCER47.CEL | GSE163722 | Immune cluster | Immune | Immune | 0.300 | 2951.889 | Immune-activated | Non-responder |
| DJN_005_HuGene2.CEL | E-MTAB-6877 | Immune cluster | Epithelial/IFN response | Immune | 0.227 | 1447.539 | Immune-activated | Responder |
| GSM4984483_MCER135_HuGene-1_1-st-v1_F10.CEL | GSE163722 | Immune cluster | Immune | Immune | 0.256 | 1938.226 | Immune-activated | Responder |
| GSM1235041 | GSE51024 | Immune cluster | Immune | Immune | 0.388 | 2649.621 | Immune-activated | Non-responder |
| GSM4984497_MCER149_HuGene-1_1-st-v1_C08.CEL | GSE163722 | Immune cluster | Epithelial/IFN response | Immune | 0.189 | 1906.845 | Immune-activated | Non-responder |
| GSM725570.CEL | MSKCC-GSE29354 | Immune cluster | Immune | Immune | 0.387 | 3247.011 | Immune-activated | Non-responder |
| DJN_106_HuGene2.CEL | E-MTAB-6877 | Immune cluster | Immune | Immune | 0.282 | 1674.308 | Immune-activated | Responder |
| GSM4984419_MCER56_HuGene-1_1-st-v1_E09.CEL | GSE163722 | Immune cluster | Immune | Immune | 0.435 | 3718.801 | Immune-activated | Responder |
| GSM1235097 | GSE51024 | Immune cluster | Immune | Immune | 0.421 | 3086.475 | Immune-activated | Non-responder |
| GSM4984446_MCER86.CEL | GSE163722 | Immune cluster | Epithelial/IFN response | Immune | 0.217 | 1774.941 | Immune-activated | Responder |
| GSM1235075 | GSE51024 | Immune cluster | Immune | Immune | 0.400 | 2698.394 | Immune-activated | Responder |
| GSM725589.CEL | MSKCC-GSE29354 | Immune cluster | Immune | Immune | 0.381 | 3201.445 | Immune-activated | Non-responder |
| GSM4984487_MCER139.CEL | GSE163722 | Immune cluster | Epithelial/IFN response | Immune | 0.238 | 2057.226 | Immune-activated | Non-responder |
| GSM4984480_MCER132_HuGene-1_1-st-v1_F09.CEL | GSE163722 | Immune cluster | Immune | Immune | 0.281 | 1854.537 | Immune-activated | Non-responder |
| GSM4984378_MCER8.CEL | GSE163722 | Immune cluster | Epithelial/IFN response | Immune | 0.240 | 1997.599 | Immune-activated | Non-responder |
| GSM4984464_MCER111.CEL | GSE163722 | Immune cluster | Epithelial/IFN response | Immune | 0.202 | 2040.815 | Immune-activated | Responder |
| DJN_022_HuGene2.CEL | E-MTAB-6877 | Immune cluster | Immune | Immune | 0.327 | 2857.324 | Immune-activated | Non-responder |
| GSM4984465_MCER113.CEL | GSE163722 | Immune cluster | Epithelial/IFN response | Immune | 0.173 | 1586.653 | Immune-activated | Responder |
| DJN_019_HuGene2.CEL | E-MTAB-6877 | Immune cluster | Immune | Immune | 0.320 | 3297.620 | Immune-activated | Non-responder |
| GSM4984403_MCER39.CEL | GSE163722 | Immune cluster | Immune | Immune | 0.263 | 1873.248 | Immune-activated | Responder |
| GSM4984455_MCER99.CEL | GSE163722 | Immune cluster | Immune | Immune | 0.258 | 1973.475 | Immune-activated | Responder |
| GSM1235067 | GSE51024 | Immune cluster | Immune | Immune | 0.345 | 1807.566 | Immune-activated | Responder |
| GSM4984445_MCER85.CEL | GSE163722 | Immune cluster | Epithelial/IFN response | Immune | 0.225 | 1679.306 | Immune-activated | Responder |
| GSM49585 | GSE2549 | Immune cluster | Epithelial/IFN response | Immune | 0.233 | 1955.362 | Immune-activated | Responder |
| GSM4984485_MCER137_HuGene-1_1-st-v1_F12.CEL | GSE163722 | Immune cluster | Immune | Immune | 0.314 | 2804.011 | Immune-activated | Non-responder |
| GSM725576.CEL | MSKCC-GSE29354 | Immune cluster | Immune | Immune | 0.313 | 3071.633 | Immune-activated | Non-responder |
| DJN_090_HuGene2.CEL | E-MTAB-6877 | Immune cluster | Epithelial/IFN response | Immune | 0.216 | 1798.049 | Immune-activated | Non-responder |
| GSM4984383_MCER14.CEL | GSE163722 | Immune cluster | Epithelial/IFN response | Immune | 0.177 | 1677.565 | Immune-activated | Responder |
| GSM4984375_MCER5.CEL | GSE163722 | Immune cluster | Immune | Immune | 0.280 | 2116.980 | Immune-activated | Non-responder |
| DJN_084_HuGene2.CEL | E-MTAB-6877 | Immune cluster | ECM | Immune | 0.160 | 1682.353 | Immune-activated | Responder |
| DJN_113_HuGene2.CEL | E-MTAB-6877 | Immune cluster | Immune | Immune | 0.365 | 2701.601 | Immune-activated | Non-responder |
| GSM4984494_MCER146_HuGene-1_1-st-v1_G06.CEL | GSE163722 | Immune cluster | Cell cycle | Immune | 0.229 | 1400.822 | Immune-activated | Non-responder |
| GSM725588.CEL | MSKCC-GSE29354 | Immune cluster | Immune | Immune | 0.388 | 3260.453 | Immune-activated | Non-responder |
| DJN_006_HuGene2.CEL | E-MTAB-6877 | Immune cluster | Epithelial/IFN response | Immune | 0.165 | 1763.038 | Immune-activated | Responder |
| GSM1235017 | GSE51024 | Immune cluster | Immune | Immune | 0.414 | 2844.482 | Immune-activated | Non-responder |
| GSM4984399_MCER33.CEL | GSE163722 | Immune cluster | Immune | Immune | 0.401 | 2387.405 | Immune-activated | Non-responder |
| GSM49568 | GSE2549 | Immune cluster | Immune | Immune | 0.272 | 1722.077 | Immune-activated | Responder |
| DJN_108_HuGene2.CEL | E-MTAB-6877 | Immune cluster | Epithelial/IFN response | Immune | 0.235 | 1764.435 | Immune-activated | Non-responder |
| GSM4984402_MCER37.CEL | GSE163722 | Immune cluster | Immune | Immune | 0.290 | 2093.650 | Immune-activated | Responder |
| GSM1235073 | GSE51024 | Immune cluster | Immune | Immune | 0.293 | 1450.423 | Immune-activated | Responder |
| GSM4984386_MCER18_HuGene-1_1-st-v1_H06.CEL | GSE163722 | Immune cluster | Epithelial/IFN response | Immune | 0.219 | 1784.785 | Immune-activated | Non-responder |
| GSM1235063 | GSE51024 | Immune cluster | Immune | Immune | 0.353 | 1775.909 | Immune-activated | Responder |
| GSM4984489_MCER141.CEL | GSE163722 | Immune cluster | ECM | Immune | 0.201 | 1488.782 | Immune-activated | Non-responder |
| GSM1235062 | GSE51024 | Immune cluster | Immune | Immune | 0.328 | 1579.914 | Immune-activated | Responder |
| GSM4984432_MCER70.CEL | GSE163722 | Immune cluster | Immune | Immune | 0.294 | 1533.732 | Immune-activated | Responder |
| GSM4984462_MCER109.CEL | GSE163722 | Immune cluster | Immune | Immune | 0.323 | 2677.477 | Immune-activated | Non-responder |
| DJN_101_HuGene2.CEL | E-MTAB-6877 | Immune cluster | Immune | Immune | 0.396 | 2295.984 | Immune-activated | Non-responder |
| GSM49599 | GSE2549 | Immune cluster | Epithelial/IFN response | Immune | 0.193 | 1326.051 | Immune-activated | Responder |
| DJN_031_HuGene2.CEL | E-MTAB-6877 | Immune cluster | Immune | Immune | 0.283 | 1999.809 | Immune-activated | Responder |
| GSM4984392_MCER25_HuGene-1_1-st-v1_G05.CEL | GSE163722 | Immune cluster | Immune | Immune | 0.235 | 1909.319 | Immune-activated | Responder |
| GSM4984377_MCER7.ga.cel | GSE163722 | Immune cluster | Epithelial/IFN response | Immune | 0.175 | 2152.997 | Immune-activated | Responder |
| GSM725604.CEL | MSKCC-GSE29354 | Immune cluster | Cell cycle | Immune | 0.216 | 1471.222 | Immune-activated | Non-responder |
| DJN_089_HuGene2.CEL | E-MTAB-6877 | Immune cluster | Immune | Immune | 0.239 | 1567.063 | Immune-activated | Non-responder |
| GSM4984387_MCER19_HuGene-1_1-st-v1_H07.CEL | GSE163722 | Immune cluster | Immune | Immune | 0.295 | 1573.965 | Immune-activated | Responder |
| GSM4984384_MCER15.CEL | GSE163722 | Immune cluster | Epithelial/IFN response | Immune | 0.234 | 1806.467 | Immune-activated | Non-responder |
| GSM725566.CEL | MSKCC-GSE29354 | Non-immune cluster | Epithelial/IFN response | Non-immune | 0.163 | 1017.178 | Non-immune | Responder |
| GSM725567.CEL | MSKCC-GSE29354 | Non-immune cluster | Cell cycle | Non-immune | 0.183 | 175.050 | Non-immune | Non-responder |
| GSM725568.CEL | MSKCC-GSE29354 | Non-immune cluster | Immune | Non-immune | 0.241 | 1270.839 | Non-immune | Responder |
| GSM725571.CEL | MSKCC-GSE29354 | Non-immune cluster | ECM | Non-immune | 0.165 | 453.132 | Non-immune | Non-responder |
| GSM725573.CEL | MSKCC-GSE29354 | Non-immune cluster | ECM | Non-immune | 0.178 | 962.187 | Non-immune | Responder |
| GSM725574.CEL | MSKCC-GSE29354 | Non-immune cluster | Cell cycle | Non-immune | 0.252 | 510.486 | Non-immune | Non-responder |
| GSM725575.CEL | MSKCC-GSE29354 | Non-immune cluster | Epithelial/IFN response | Non-immune | 0.185 | 693.365 | Non-immune | Responder |
| GSM725577.CEL | MSKCC-GSE29354 | Non-immune cluster | Epithelial/IFN response | Non-immune | 0.112 | 363.226 | Non-immune | Responder |
| GSM725578.CEL | MSKCC-GSE29354 | Immune cluster | Immune | Non-immune | 0.311 | 1480.353 | Non-immune | Non-responder |
| GSM725579.CEL | MSKCC-GSE29354 | Non-immune cluster | Epithelial/IFN response | Non-immune | 0.172 | 825.901 | Non-immune | Non-responder |
| GSM725580.CEL | MSKCC-GSE29354 | Non-immune cluster | Cell cycle | Non-immune | 0.185 | -46.080 | Non-immune | Non-responder |
| GSM725581.CEL | MSKCC-GSE29354 | Non-immune cluster | Cell cycle | Non-immune | 0.164 | 1120.887 | Non-immune | Non-responder |
| GSM725582.CEL | MSKCC-GSE29354 | Non-immune cluster | Cell cycle | Non-immune | 0.216 | 816.050 | Non-immune | Non-responder |
| GSM725583.CEL | MSKCC-GSE29354 | Non-immune cluster | Immune | Non-immune | 0.237 | 1272.349 | Non-immune | Non-responder |
| GSM725584.CEL | MSKCC-GSE29354 | Non-immune cluster | Epithelial/IFN response | Non-immune | 0.138 | 413.845 | Non-immune | Non-responder |
| GSM725585.CEL | MSKCC-GSE29354 | Non-immune cluster | Cell cycle | Non-immune | 0.168 | 560.873 | Non-immune | Non-responder |
| GSM725586.CEL | MSKCC-GSE29354 | Non-immune cluster | ECM | Non-immune | 0.146 | 786.316 | Non-immune | Non-responder |
| GSM725590.CEL | MSKCC-GSE29354 | Non-immune cluster | ECM | Non-immune | 0.129 | 451.263 | Non-immune | Non-responder |
| GSM725592.CEL | MSKCC-GSE29354 | Non-immune cluster | Cell cycle | Non-immune | 0.179 | -5.319 | Non-immune | Non-responder |
| GSM725593.CEL | MSKCC-GSE29354 | Non-immune cluster | Epithelial/IFN response | Non-immune | 0.122 | 596.823 | Non-immune | Non-responder |
| GSM725596.CEL | MSKCC-GSE29354 | Non-immune cluster | Epithelial/IFN response | Non-immune | 0.192 | 1139.715 | Non-immune | Responder |
| GSM725597.CEL | MSKCC-GSE29354 | Non-immune cluster | Cell cycle | Non-immune | 0.182 | 170.619 | Non-immune | Non-responder |
| GSM725598.CEL | MSKCC-GSE29354 | Non-immune cluster | Epithelial/IFN response | Non-immune | 0.143 | 968.739 | Non-immune | Responder |
| GSM725599.CEL | MSKCC-GSE29354 | Non-immune cluster | Epithelial/IFN response | Non-immune | 0.143 | 1063.605 | Non-immune | Responder |
| GSM725601.CEL | MSKCC-GSE29354 | Non-immune cluster | Cell cycle | Non-immune | 0.241 | -37.112 | Non-immune | Responder |
| GSM725602.CEL | MSKCC-GSE29354 | Immune cluster | Cell cycle | Non-immune | 0.225 | 1207.362 | Non-immune | Responder |
| GSM725603.CEL | MSKCC-GSE29354 | Non-immune cluster | Epithelial/IFN response | Non-immune | 0.151 | 730.438 | Non-immune | Non-responder |
| GSM725605.CEL | MSKCC-GSE29354 | Non-immune cluster | ECM | Non-immune | 0.188 | 883.167 | Non-immune | Non-responder |
| GSM725607.CEL | MSKCC-GSE29354 | Non-immune cluster | ECM | Non-immune | 0.194 | 1513.949 | Non-immune | Non-responder |
| GSM725609.CEL | MSKCC-GSE29354 | Non-immune cluster | Epithelial/IFN response | Non-immune | 0.101 | 550.816 | Non-immune | Non-responder |
| GSM725610.CEL | MSKCC-GSE29354 | Non-immune cluster | Epithelial/IFN response | Non-immune | 0.152 | 867.464 | Non-immune | Non-responder |
| GSM725611.CEL | MSKCC-GSE29354 | Non-immune cluster | ECM | Non-immune | 0.130 | 352.227 | Non-immune | Non-responder |
| GSM725612.CEL | MSKCC-GSE29354 | Non-immune cluster | Immune | Non-immune | 0.274 | 1347.866 | Non-immune | Non-responder |
| GSM725613.CEL | MSKCC-GSE29354 | Non-immune cluster | Epithelial/IFN response | Non-immune | 0.137 | 1494.962 | Non-immune | Responder |
| GSM725614.CEL | MSKCC-GSE29354 | Non-immune cluster | Cell cycle | Non-immune | 0.224 | 25.613 | Non-immune | Non-responder |
| GSM725616.CEL | MSKCC-GSE29354 | Non-immune cluster | Cell cycle | Non-immune | 0.216 | 86.071 | Non-immune | Non-responder |
| GSM725618.CEL | MSKCC-GSE29354 | Non-immune cluster | Epithelial/IFN response | Non-immune | 0.194 | 1303.514 | Non-immune | Responder |
| GSM49564 | GSE2549 | Non-immune cluster | Cell cycle | Non-immune | 0.253 | 769.395 | Non-immune | Non-responder |
| GSM49567 | GSE2549 | Non-immune cluster | Cell cycle | Non-immune | 0.218 | 1293.081 | Non-immune | Responder |
| GSM49569 | GSE2549 | Non-immune cluster | Cell cycle | Non-immune | 0.197 | 614.768 | Non-immune | Non-responder |
| GSM49571 | GSE2549 | Non-immune cluster | ECM | Non-immune | 0.183 | 1008.366 | Non-immune | Non-responder |
| GSM49572 | GSE2549 | Non-immune cluster | Epithelial/IFN response | Non-immune | 0.156 | 1067.681 | Non-immune | Responder |
| GSM49573 | GSE2549 | Immune cluster | ECM | Non-immune | 0.176 | 1323.376 | Non-immune | Responder |
| GSM49574 | GSE2549 | Non-immune cluster | Epithelial/IFN response | Non-immune | 0.137 | 832.294 | Non-immune | Responder |
| GSM49575 | GSE2549 | Non-immune cluster | Epithelial/IFN response | Non-immune | 0.189 | 840.103 | Non-immune | Responder |
| GSM49576 | GSE2549 | Non-immune cluster | Cell cycle | Non-immune | 0.194 | 309.916 | Non-immune | Non-responder |
| GSM49577 | GSE2549 | Non-immune cluster | ECM | Non-immune | 0.150 | 724.495 | Non-immune | Non-responder |
| GSM49579 | GSE2549 | Non-immune cluster | Cell cycle | Non-immune | 0.224 | 1088.074 | Non-immune | Non-responder |
| GSM49580 | GSE2549 | Non-immune cluster | Cell cycle | Non-immune | 0.220 | 1066.170 | Non-immune | Non-responder |
| GSM49581 | GSE2549 | Non-immune cluster | Cell cycle | Non-immune | 0.175 | 657.158 | Non-immune | Non-responder |
| GSM49582 | GSE2549 | Non-immune cluster | Epithelial/IFN response | Non-immune | 0.175 | 673.338 | Non-immune | Responder |
| GSM49584 | GSE2549 | Non-immune cluster | Epithelial/IFN response | Non-immune | 0.201 | 1200.809 | Non-immune | Responder |
| GSM49586 | GSE2549 | Non-immune cluster | ECM | Non-immune | 0.184 | 1079.747 | Non-immune | Non-responder |
| GSM49587 | GSE2549 | Non-immune cluster | Epithelial/IFN response | Non-immune | 0.171 | 1156.602 | Non-immune | Responder |
| GSM49588 | GSE2549 | Non-immune cluster | Epithelial/IFN response | Non-immune | 0.200 | 1038.345 | Non-immune | Non-responder |
| GSM49590 | GSE2549 | Non-immune cluster | Cell cycle | Non-immune | 0.237 | 720.440 | Non-immune | Non-responder |
| GSM49591 | GSE2549 | Non-immune cluster | Epithelial/IFN response | Non-immune | 0.173 | 1261.861 | Non-immune | Non-responder |
| GSM49592 | GSE2549 | Non-immune cluster | Epithelial/IFN response | Non-immune | 0.167 | 1059.523 | Non-immune | Responder |
| GSM49593 | GSE2549 | Non-immune cluster | Cell cycle | Non-immune | 0.152 | 32.213 | Non-immune | Non-responder |
| GSM49596 | GSE2549 | Non-immune cluster | Cell cycle | Non-immune | 0.175 | 664.516 | Non-immune | Non-responder |
| GSM49598 | GSE2549 | Non-immune cluster | Cell cycle | Non-immune | 0.179 | 376.104 | Non-immune | Non-responder |
| GSM49600 | GSE2549 | Non-immune cluster | Cell cycle | Non-immune | 0.232 | 832.825 | Non-immune | Responder |
| GSM49603 | GSE2549 | Immune cluster | Immune | Non-immune | 0.240 | 1443.422 | Non-immune | Non-responder |
| GSM1235019 | GSE51024 | Non-immune cluster | Epithelial/IFN response | Non-immune | 0.159 | 1268.085 | Non-immune | Responder |
| GSM1235025 | GSE51024 | Non-immune cluster | Cell cycle | Non-immune | 0.184 | 331.718 | Non-immune | Non-responder |
| GSM1235027 | GSE51024 | Non-immune cluster | ECM | Non-immune | 0.183 | 859.528 | Non-immune | Non-responder |
| GSM1235029 | GSE51024 | Non-immune cluster | Epithelial/IFN response | Non-immune | 0.133 | 1038.855 | Non-immune | Non-responder |
| GSM1235031 | GSE51024 | Non-immune cluster | Epithelial/IFN response | Non-immune | 0.079 | 81.680 | Non-immune | Non-responder |
| GSM1235033 | GSE51024 | Non-immune cluster | Epithelial/IFN response | Non-immune | 0.154 | 1147.243 | Non-immune | Responder |
| GSM1235035 | GSE51024 | Non-immune cluster | ECM | Non-immune | 0.181 | 858.415 | Non-immune | Non-responder |
| GSM1235039 | GSE51024 | Non-immune cluster | ECM | Non-immune | 0.201 | 1115.686 | Non-immune | Non-responder |
| GSM1235043 | GSE51024 | Non-immune cluster | Cell cycle | Non-immune | 0.223 | 1289.367 | Non-immune | Responder |
| GSM1235045 | GSE51024 | Non-immune cluster | Cell cycle | Non-immune | 0.288 | 1013.511 | Non-immune | Non-responder |
| GSM1235049 | GSE51024 | Non-immune cluster | Cell cycle | Non-immune | 0.182 | 979.665 | Non-immune | Non-responder |
| GSM1235051 | GSE51024 | Non-immune cluster | Cell cycle | Non-immune | 0.212 | 451.286 | Non-immune | Non-responder |
| GSM1235053 | GSE51024 | Non-immune cluster | Epithelial/IFN response | Non-immune | 0.174 | 1158.671 | Non-immune | Responder |
| GSM1235055 | GSE51024 | Non-immune cluster | Cell cycle | Non-immune | 0.110 | -57.412 | Non-immune | Non-responder |
| GSM1235059 | GSE51024 | Non-immune cluster | ECM | Non-immune | 0.199 | 535.011 | Non-immune | Responder |
| GSM1235061 | GSE51024 | Non-immune cluster | Cell cycle | Non-immune | 0.140 | 114.064 | Non-immune | Non-responder |
| GSM1235065 | GSE51024 | Non-immune cluster | Cell cycle | Non-immune | 0.155 | 482.574 | Non-immune | Non-responder |
| GSM1235071 | GSE51024 | Non-immune cluster | Cell cycle | Non-immune | 0.205 | 977.565 | Non-immune | Non-responder |
| GSM1235074 | GSE51024 | Non-immune cluster | Cell cycle | Non-immune | 0.145 | 29.960 | Non-immune | Non-responder |
| GSM1235081 | GSE51024 | Non-immune cluster | ECM | Non-immune | 0.167 | 1489.323 | Non-immune | Responder |
| GSM1235083 | GSE51024 | Non-immune cluster | Cell cycle | Non-immune | 0.152 | -236.448 | Non-immune | Non-responder |
| GSM1235084 | GSE51024 | Non-immune cluster | Cell cycle | Non-immune | 0.157 | 148.578 | Non-immune | Non-responder |
| GSM1235085 | GSE51024 | Non-immune cluster | Cell cycle | Non-immune | 0.212 | 1262.884 | Non-immune | Responder |
| GSM1235092 | GSE51024 | Non-immune cluster | Epithelial/IFN response | Non-immune | 0.172 | 1243.665 | Non-immune | Responder |
| GSM1235093 | GSE51024 | Non-immune cluster | Epithelial/IFN response | Non-immune | 0.189 | 841.890 | Non-immune | Non-responder |
| GSM1235094 | GSE51024 | Non-immune cluster | ECM | Non-immune | 0.115 | 270.801 | Non-immune | Responder |
| GSM1235096 | GSE51024 | Non-immune cluster | Epithelial/IFN response | Non-immune | 0.146 | 423.063 | Non-immune | Responder |
| GSM1235098 | GSE51024 | Non-immune cluster | ECM | Non-immune | 0.124 | 137.710 | Non-immune | Non-responder |
| GSM1235099 | GSE51024 | Non-immune cluster | ECM | Non-immune | 0.122 | 749.895 | Non-immune | Non-responder |
| GSM1235101 | GSE51024 | Non-immune cluster | Cell cycle | Non-immune | 0.238 | 1003.052 | Non-immune | Non-responder |
| GSM1235107 | GSE51024 | Non-immune cluster | Epithelial/IFN response | Non-immune | 0.127 | 1152.750 | Non-immune | Responder |
| GSM1235109 | GSE51024 | Non-immune cluster | ECM | Non-immune | 0.189 | 1250.396 | Non-immune | Non-responder |
| GSM1235111 | GSE51024 | Non-immune cluster | ECM | Non-immune | 0.155 | 771.577 | Non-immune | Non-responder |
| GSM4984371_MCER1.CEL | GSE163722 | Non-immune cluster | Epithelial/IFN response | Non-immune | 0.105 | -71.257 | Non-immune | Non-responder |
| GSM4984372_MCER2.ga.cel | GSE163722 | Non-immune cluster | ECM | Non-immune | 0.127 | 1523.423 | Non-immune | Responder |
| GSM4984373_MCER3.CEL | GSE163722 | Non-immune cluster | Cell cycle | Non-immune | 0.166 | -27.416 | Non-immune | Non-responder |
| GSM4984374_MCER4.CEL | GSE163722 | Non-immune cluster | Cell cycle | Non-immune | 0.190 | 1153.550 | Non-immune | Responder |
| GSM4984376_MCER6.CEL | GSE163722 | Immune cluster | Epithelial/IFN response | Non-immune | 0.211 | 1186.183 | Non-immune | Non-responder |
| GSM4984379_MCER9.CEL | GSE163722 | Non-immune cluster | Immune | Non-immune | 0.221 | 999.915 | Non-immune | Responder |
| GSM4984380_MCER10.CEL | GSE163722 | Non-immune cluster | Cell cycle | Non-immune | 0.244 | 1159.824 | Non-immune | Non-responder |
| GSM4984381_MCER11.ga.cel | GSE163722 | Non-immune cluster | ECM | Non-immune | 0.119 | 1205.734 | Non-immune | Responder |
| GSM4984382_MCER13.CEL | GSE163722 | Non-immune cluster | Cell cycle | Non-immune | 0.264 | 375.243 | Non-immune | Non-responder |
| GSM4984389_MCER21_HuGene-1_1-st-v1_H08.CEL | GSE163722 | Non-immune cluster | Cell cycle | Non-immune | 0.194 | 523.578 | Non-immune | Non-responder |
| GSM4984390_MCER22_HuGene-1_1-st-v1_H09.CEL | GSE163722 | Non-immune cluster | Cell cycle | Non-immune | 0.181 | 29.101 | Non-immune | Non-responder |
| GSM4984391_MCER23_HuGene-1_1-st-v1_H10.CEL | GSE163722 | Non-immune cluster | Cell cycle | Non-immune | 0.146 | -71.121 | Non-immune | Non-responder |
| GSM4984394_MCER28.CEL | GSE163722 | Non-immune cluster | Cell cycle | Non-immune | 0.161 | 115.572 | Non-immune | Non-responder |
| GSM4984395_MCER29.CEL | GSE163722 | Non-immune cluster | Cell cycle | Non-immune | 0.201 | 603.187 | Non-immune | Non-responder |
| GSM4984397_MCER31_HuGene-1_1-st-v1_H12.CEL | GSE163722 | Non-immune cluster | Cell cycle | Non-immune | 0.137 | 100.770 | Non-immune | Non-responder |
| GSM4984398_MCER32.CEL | GSE163722 | Non-immune cluster | Cell cycle | Non-immune | 0.185 | 541.849 | Non-immune | Non-responder |
| GSM4984400_MCER34.CEL | GSE163722 | Non-immune cluster | Cell cycle | Non-immune | 0.184 | 455.207 | Non-immune | Non-responder |
| GSM4984404_MCER40.CEL | GSE163722 | Non-immune cluster | Epithelial/IFN response | Non-immune | 0.148 | 930.585 | Non-immune | Responder |
| GSM4984405_MCER41.CEL | GSE163722 | Non-immune cluster | Cell cycle | Non-immune | 0.185 | -1499.762 | Non-immune | Non-responder |
| GSM4984406_MCER41.ga.cel | GSE163722 | Non-immune cluster | Cell cycle | Non-immune | 0.179 | -1582.602 | Non-immune | Non-responder |
| GSM4984408_MCER43_HuGene-1_1-st-v1_G11.CEL | GSE163722 | Non-immune cluster | Cell cycle | Non-immune | 0.201 | 805.532 | Non-immune | Non-responder |
| GSM4984409_MCER45_HuGene-1_1-st-v1_G12.CEL | GSE163722 | Non-immune cluster | Epithelial/IFN response | Non-immune | 0.139 | 506.234 | Non-immune | Responder |
| GSM4984410_MCER46.CEL | GSE163722 | Non-immune cluster | Epithelial/IFN response | Non-immune | 0.194 | 1134.711 | Non-immune | Responder |
| GSM4984413_MCER49.CEL | GSE163722 | Non-immune cluster | Epithelial/IFN response | Non-immune | 0.137 | 657.931 | Non-immune | Responder |
| GSM4984415_MCER51.CEL | GSE163722 | Non-immune cluster | Cell cycle | Non-immune | 0.228 | 958.794 | Non-immune | Non-responder |
| GSM4984416_MCER52.CEL | GSE163722 | Non-immune cluster | ECM | Non-immune | 0.208 | 1009.790 | Non-immune | Non-responder |
| GSM4984417_MCER53.CEL | GSE163722 | Non-immune cluster | Cell cycle | Non-immune | 0.220 | 31.176 | Non-immune | Non-responder |
| GSM4984418_MCER54.CEL | GSE163722 | Immune cluster | Immune | Non-immune | 0.245 | 1291.922 | Non-immune | Non-responder |
| GSM4984420_MCER57_HuGene-1_1-st-v1_E10.CEL | GSE163722 | Non-immune cluster | Cell cycle | Non-immune | 0.119 | -968.627 | Non-immune | Non-responder |
| GSM4984421_MCER59.CEL | GSE163722 | Non-immune cluster | Epithelial/IFN response | Non-immune | 0.143 | 277.230 | Non-immune | Non-responder |
| GSM4984422_MCER60.CEL | GSE163722 | Non-immune cluster | Epithelial/IFN response | Non-immune | 0.111 | -15.167 | Non-immune | Non-responder |
| GSM4984423_MCER61.CEL | GSE163722 | Non-immune cluster | Epithelial/IFN response | Non-immune | 0.116 | 645.782 | Non-immune | Responder |
| GSM4984424_MCER62.CEL | GSE163722 | Non-immune cluster | Cell cycle | Non-immune | 0.137 | 738.846 | Non-immune | Non-responder |
| GSM4984426_MCER64.CEL | GSE163722 | Non-immune cluster | Epithelial/IFN response | Non-immune | 0.077 | 296.122 | Non-immune | Responder |
| GSM4984427_MCER65.CEL | GSE163722 | Immune cluster | Immune | Non-immune | 0.271 | 1261.286 | Non-immune | Non-responder |
| GSM4984428_MCER66.CEL | GSE163722 | Non-immune cluster | Cell cycle | Non-immune | 0.188 | -136.662 | Non-immune | Non-responder |
| GSM4984433_MCER71_HuGene-1_1-st-v1_G02.CEL | GSE163722 | Non-immune cluster | Cell cycle | Non-immune | 0.130 | -292.967 | Non-immune | Non-responder |
| GSM4984434_MCER73_HuGene-1_1-st-v1_G03.CEL | GSE163722 | Non-immune cluster | Cell cycle | Non-immune | 0.137 | 318.582 | Non-immune | Non-responder |
| GSM4984435_MCER74.CEL | GSE163722 | Immune cluster | Immune | Non-immune | 0.297 | 1180.469 | Non-immune | Responder |
| GSM4984437_MCER77.CEL | GSE163722 | Non-immune cluster | Epithelial/IFN response | Non-immune | 0.126 | 1300.295 | Non-immune | Responder |
| GSM4984439_MCER80.CEL | GSE163722 | Non-immune cluster | ECM | Non-immune | 0.109 | 1061.605 | Non-immune | Responder |
| GSM4984440_MCER81.CEL | GSE163722 | Non-immune cluster | Epithelial/IFN response | Non-immune | 0.170 | 1024.680 | Non-immune | Responder |
| GSM4984441_MCER81-2.CEL | GSE163722 | Non-immune cluster | Epithelial/IFN response | Non-immune | 0.165 | 1076.662 | Non-immune | Responder |
| GSM4984444_MCER84.CEL | GSE163722 | Non-immune cluster | Epithelial/IFN response | Non-immune | 0.178 | 843.771 | Non-immune | Responder |
| GSM4984448_MCER88.CEL | GSE163722 | Non-immune cluster | Epithelial/IFN response | Non-immune | 0.152 | 1181.385 | Non-immune | Non-responder |
| GSM4984449_MCER91.CEL | GSE163722 | Non-immune cluster | Cell cycle | Non-immune | 0.227 | 690.939 | Non-immune | Non-responder |
| GSM4984450_MCER92.CEL | GSE163722 | Non-immune cluster | Cell cycle | Non-immune | 0.186 | 846.521 | Non-immune | Non-responder |
| GSM4984451_MCER93.CEL | GSE163722 | Non-immune cluster | Cell cycle | Non-immune | 0.182 | 954.179 | Non-immune | Responder |
| GSM4984452_MCER94.CEL | GSE163722 | Non-immune cluster | Epithelial/IFN response | Non-immune | 0.209 | 1267.958 | Non-immune | Responder |
| GSM4984453_MCER97.CEL | GSE163722 | Non-immune cluster | Cell cycle | Non-immune | 0.225 | 777.340 | Non-immune | Non-responder |
| GSM4984454_MCER98.CEL | GSE163722 | Non-immune cluster | Epithelial/IFN response | Non-immune | 0.183 | 1208.021 | Non-immune | Responder |
| GSM4984457_MCER103.CEL | GSE163722 | Non-immune cluster | Cell cycle | Non-immune | 0.241 | 92.791 | Non-immune | Non-responder |
| GSM4984461_MCER108.CEL | GSE163722 | Non-immune cluster | ECM | Non-immune | 0.194 | 1129.244 | Non-immune | Responder |
| GSM4984466_MCER114.CEL | GSE163722 | Non-immune cluster | Epithelial/IFN response | Non-immune | 0.141 | 914.472 | Non-immune | Responder |
| GSM4984468_MCER118.CEL | GSE163722 | Non-immune cluster | Cell cycle | Non-immune | 0.223 | 1229.443 | Non-immune | Responder |
| GSM4984469_MCER119.CEL | GSE163722 | Non-immune cluster | Epithelial/IFN response | Non-immune | 0.205 | 642.414 | Non-immune | Responder |
| GSM4984472_MCER123.CEL | GSE163722 | Non-immune cluster | Cell cycle | Non-immune | 0.142 | 113.224 | Non-immune | Non-responder |
| GSM4984473_MCER124.CEL | GSE163722 | Non-immune cluster | Cell cycle | Non-immune | 0.229 | 780.707 | Non-immune | Non-responder |
| GSM4984474_MCER125.CEL | GSE163722 | Non-immune cluster | Epithelial/IFN response | Non-immune | 0.116 | 432.586 | Non-immune | Responder |
| GSM4984476_MCER128.CEL | GSE163722 | Non-immune cluster | Cell cycle | Non-immune | 0.257 | -11.944 | Non-immune | Non-responder |
| GSM4984477_MCER129_HuGene-1_1-st-v1_F06.CEL | GSE163722 | Non-immune cluster | ECM | Non-immune | 0.208 | 1397.536 | Non-immune | Non-responder |
| GSM4984478_MCER130_HuGene-1_1-st-v1_F07.CEL | GSE163722 | Non-immune cluster | Cell cycle | Non-immune | 0.172 | 102.132 | Non-immune | Non-responder |
| GSM4984479_MCER131_HuGene-1_1-st-v1_F08.CEL | GSE163722 | Non-immune cluster | Cell cycle | Non-immune | 0.246 | 235.905 | Non-immune | Non-responder |
| GSM4984481_MCER133_HuGene-1_1-st-v1_H01.CEL | GSE163722 | Non-immune cluster | ECM | Non-immune | 0.188 | 636.128 | Non-immune | Non-responder |
| GSM4984482_MCER134_HuGene-1_1-st-v1_C07.CEL | GSE163722 | Non-immune cluster | ECM | Non-immune | 0.151 | 954.682 | Non-immune | Non-responder |
| GSM4984486_MCER138.CEL | GSE163722 | Non-immune cluster | ECM | Non-immune | 0.153 | 758.772 | Non-immune | Non-responder |
| GSM4984488_MCER140.CEL | GSE163722 | Non-immune cluster | Cell cycle | Non-immune | 0.252 | 486.479 | Non-immune | Non-responder |
| GSM4984491_MCER143.CEL | GSE163722 | Non-immune cluster | Epithelial/IFN response | Non-immune | 0.210 | 869.425 | Non-immune | Responder |
| GSM4984492_MCER144_HuGene-1_1-st-v1_G09.CEL | GSE163722 | Non-immune cluster | Cell cycle | Non-immune | 0.197 | 442.384 | Non-immune | Non-responder |
| GSM4984493_MCER145_HuGene-1_1-st-v1_G10.CEL | GSE163722 | Non-immune cluster | Cell cycle | Non-immune | 0.202 | 1209.036 | Non-immune | Non-responder |
| GSM4984498_MCER150_HuGene-1_1-st-v1_H02.CEL | GSE163722 | Non-immune cluster | Cell cycle | Non-immune | 0.241 | 821.803 | Non-immune | Non-responder |
| GSM4984499_MCER152_HuGene-1_1-st-v1_A04.CEL | GSE163722 | Non-immune cluster | Cell cycle | Non-immune | 0.151 | 46.317 | Non-immune | Non-responder |
| GSM4984500_MCER153_HuGene-1_1-st-v1_H03.CEL | GSE163722 | Immune cluster | Epithelial/IFN response | Non-immune | 0.204 | 1373.991 | Non-immune | Non-responder |
| GSM4984501_MCER154_HuGene-1_1-st-v1_H04.CEL | GSE163722 | Non-immune cluster | Cell cycle | Non-immune | 0.127 | -110.825 | Non-immune | Non-responder |
| DJN_098_HuGene2.CEL | E-MTAB-6877 | Non-immune cluster | Epithelial/IFN response | Non-immune | 0.092 | 586.547 | Non-immune | Responder |
| DJN_114_HuGene2.CEL | E-MTAB-6877 | Non-immune cluster | ECM | Non-immune | 0.148 | 1070.226 | Non-immune | Responder |
| DJN_107_HuGene2.CEL | E-MTAB-6877 | Immune cluster | Immune | Non-immune | 0.311 | 1203.410 | Non-immune | Non-responder |
| DJN_030_HuGene2.CEL | E-MTAB-6877 | Non-immune cluster | ECM | Non-immune | 0.176 | 1363.465 | Non-immune | Non-responder |
| DJN_116_HuGene2.CEL | E-MTAB-6877 | Non-immune cluster | Cell cycle | Non-immune | 0.204 | 1227.666 | Non-immune | Non-responder |
| DJN_093_HuGene2.CEL | E-MTAB-6877 | Non-immune cluster | Cell cycle | Non-immune | 0.140 | 214.032 | Non-immune | Non-responder |
| DJN_117_HuGene2.CEL | E-MTAB-6877 | Non-immune cluster | ECM | Non-immune | 0.147 | 1242.893 | Non-immune | Responder |
| DJN_096_HuGene2.CEL | E-MTAB-6877 | Non-immune cluster | ECM | Non-immune | 0.184 | 1125.504 | Non-immune | Non-responder |
| DJN_124_HuGene2.CEL | E-MTAB-6877 | Non-immune cluster | Cell cycle | Non-immune | 0.175 | 549.620 | Non-immune | Non-responder |
| DJN_025_HuGene2.CEL | E-MTAB-6877 | Non-immune cluster | Cell cycle | Non-immune | 0.152 | -391.243 | Non-immune | Non-responder |
| DJN_029_HuGene2.CEL | E-MTAB-6877 | Non-immune cluster | Epithelial/IFN response | Non-immune | 0.141 | 650.106 | Non-immune | Non-responder |
| DJN_085_HuGene2.CEL | E-MTAB-6877 | Non-immune cluster | Epithelial/IFN response | Non-immune | 0.080 | 412.730 | Non-immune | Non-responder |
| DJN_088_HuGene2.CEL | E-MTAB-6877 | Non-immune cluster | Epithelial/IFN response | Non-immune | 0.146 | 690.154 | Non-immune | Non-responder |
| DJN_097_HuGene2.CEL | E-MTAB-6877 | Non-immune cluster | Epithelial/IFN response | Non-immune | 0.118 | 609.289 | Non-immune | Non-responder |
| DJN_010_HuGene2.CEL | E-MTAB-6877 | Non-immune cluster | Cell cycle | Non-immune | 0.170 | 245.799 | Non-immune | Non-responder |
| DJN_027_HuGene2.CEL | E-MTAB-6877 | Non-immune cluster | Cell cycle | Non-immune | 0.238 | 185.147 | Non-immune | Non-responder |
| DJN_016_HuGene2.CEL | E-MTAB-6877 | Non-immune cluster | Cell cycle | Non-immune | 0.202 | -370.102 | Non-immune | Non-responder |
| DJN_104_HuGene2.CEL | E-MTAB-6877 | Non-immune cluster | Epithelial/IFN response | Non-immune | 0.150 | 1010.823 | Non-immune | Non-responder |
| DJN_111_HuGene2.CEL | E-MTAB-6877 | Non-immune cluster | ECM | Non-immune | 0.132 | 1413.985 | Non-immune | Responder |
| DJN_007_HuGene2.CEL | E-MTAB-6877 | Non-immune cluster | Epithelial/IFN response | Non-immune | 0.111 | 1122.865 | Non-immune | Responder |
| DJN_024_HuGene2.CEL | E-MTAB-6877 | Non-immune cluster | Cell cycle | Non-immune | 0.128 | -116.135 | Non-immune | Non-responder |
| DJN_091_HuGene2.CEL | E-MTAB-6877 | Non-immune cluster | Epithelial/IFN response | Non-immune | 0.093 | 411.347 | Non-immune | Non-responder |
| DJN_120_HuGene2.CEL | E-MTAB-6877 | Non-immune cluster | Cell cycle | Non-immune | 0.176 | 572.735 | Non-immune | Non-responder |
| DJN_112_HuGene2.CEL | E-MTAB-6877 | Non-immune cluster | ECM | Non-immune | 0.187 | 1048.930 | Non-immune | Non-responder |
| DJN_012_HuGene2.CEL | E-MTAB-6877 | Non-immune cluster | Cell cycle | Non-immune | 0.120 | -162.255 | Non-immune | Non-responder |
| DJN_095_HuGene2.CEL | E-MTAB-6877 | Non-immune cluster | ECM | Non-immune | 0.119 | 409.289 | Non-immune | Non-responder |
| DJN_017_HuGene2.CEL | E-MTAB-6877 | Non-immune cluster | Epithelial/IFN response | Non-immune | 0.132 | 888.982 | Non-immune | Non-responder |
| DJN_015_HuGene2.CEL | E-MTAB-6877 | Non-immune cluster | Epithelial/IFN response | Non-immune | 0.187 | 1452.489 | Non-immune | Responder |
| DJN_103_HuGene2.CEL | E-MTAB-6877 | Non-immune cluster | Epithelial/IFN response | Non-immune | 0.138 | 1368.115 | Non-immune | Responder |
| DJN_013_HuGene2.CEL | E-MTAB-6877 | Non-immune cluster | Epithelial/IFN response | Non-immune | 0.134 | 1344.711 | Non-immune | Responder |
| DJN_094_HuGene2.CEL | E-MTAB-6877 | Non-immune cluster | Cell cycle | Non-immune | 0.235 | 748.205 | Non-immune | Non-responder |
| DJN_100_HuGene2.CEL | E-MTAB-6877 | Non-immune cluster | Cell cycle | Non-immune | 0.143 | 309.953 | Non-immune | Non-responder |
| DJN_082_HuGene2.CEL | E-MTAB-6877 | Immune cluster | Cell cycle | Non-immune | 0.220 | 962.369 | Non-immune | Non-responder |
| DJN_023_HuGene2.CEL | E-MTAB-6877 | Non-immune cluster | Cell cycle | Non-immune | 0.202 | 934.138 | Non-immune | Non-responder |
| DJN_008_HuGene2.CEL | E-MTAB-6877 | Non-immune cluster | Cell cycle | Non-immune | 0.317 | 519.393 | Non-immune | Non-responder |
| DJN_121_HuGene2.CEL | E-MTAB-6877 | Non-immune cluster | Cell cycle | Non-immune | 0.313 | 567.127 | Non-immune | Non-responder |
| DJN_109_HuGene2.CEL | E-MTAB-6877 | Non-immune cluster | Immune | Non-immune | 0.289 | 985.338 | Non-immune | Non-responder |
| DJN_028_HuGene2.CEL | E-MTAB-6877 | Non-immune cluster | Cell cycle | Non-immune | 0.108 | -764.387 | Non-immune | Non-responder |
