# Supplementary material for: Unraveling tumor microenvironment heterogeneity in malignant pleural mesothelioma identifies biologically distinct immune subtypes enabling prognosis determination
Source: Front Oncol. 2022 Sep 27;12:995651. doi: 10.3389/fonc.2022.995651 (PMC9552848; doi:10.3389/fonc.2022.995651)
Supplement: Supplementary file 6 [file Table_5.docx]

| **Table 5. The distribution of three novel immune subtypes in all enrolled MPM cohorts** | | | | |
| --- | --- | --- | --- | --- |
| Dataset | No. of patients | Distribution proportion of Immune subtypes | | |
|  |  | Immune-activated | Immune- suppressed | Non-immune |
| MSKCC-GSE29354 | 52 | 5 (9.6%) | 10 (19.2%) | 37 (71.2%) |
| GSE2549 | 40 | 3 (7.5%) | 11 (27.5%） | 26 (65.0%) |
| GSE51024 | 47 | 8 (17.0%) | 6 (11.5%) | 33 (70.2%) |
| GSE163722 | 131 | 27 (20.6%) | 30 (22.9%) | 74 (56.5%) |
| E-MTAB-6877 | 63 | 12 (19.0%) | 13 (20.6%) | 38 (60.3%) |
| TCGA-MESO | 87 | 24 (27.6%) | 23 (26.4%) | 40 (46.0%) |
